# Human sensory adaptation to the ecological structure of environmental statistics

**DOI:** 10.1167/jov.24.3.3

**Published:** 2024-03-05

**Authors:** Peter Neri

**Affiliations:** 1 Laboratoire des Systèmes Perceptifs (UMR8248), École normale supérieure, PSL Research University, Paris, France

**Keywords:** virtual reality, object segmentation, statistical learning

## Abstract

Humans acquire sensory information via fast, highly specialized detectors: For example, edge detectors monitor restricted regions of visual space over timescales of 100–200 ms. Surprisingly, this study demonstrates that their operation is nevertheless shaped by the ecological consistency of slow global statistical structure in the environment. In the experiments, humans acquired feature information from brief localized elements embedded within a virtual environment. Cast shadows are important for determining the appearance and layout of the environment. When the statistical reliability of shadows was manipulated, human feature detectors implicitly adapted to these changes over minutes, adjusting their response properties to emphasize either “image-based” or “object-based” anchoring of local visual elements. More specifically, local visual operators were more firmly anchored around object representations when shadows were reliable. As shadow reliability was reduced, visual operators disengaged from objects and became anchored around image features. These results indicate that the notion of sensory adaptation must be reframed around complex statistical constructs with ecological validity. These constructs far exceed the spatiotemporal selectivity bandwidth of sensory detectors, thus demonstrating the highly integrated nature of sensory processing during natural behavior.

## Introduction

Early understanding of visual processing was dominated by the notion of specificity (Hubel, 1982): cortical neurons respond to stimuli within a restricted region of visual space (their “receptive field”), and record stimulus characteristics within the timescale of stable vision (<300 ms). The corresponding perceptual mechanisms display similar characteristics. For example, humans perceive motion via mechanisms that integrate signals over ∼100 ms (Burr & Ross, 1986), and effective manipulation of the associated percepts is often strictly local (Curran, Clifford, & Benton, 2006).

Subsequent research in both physiology and psychophysics has delineated a much more complex picture of these processes. Visual detectors may be primarily driven by localized spatiotemporal information, but the manner in which they process said information is influenced by information from other spatiotemporal regions (Bolz & Gilbert, 1986). For example, visual neurons may respond differently to an identical stimulus, depending on what came before the stimulus (Kohn, 2007) and on what is presented in the surrounding region (Allman, Miezin, & McGuinness, 1985). These effects can be complex (Roelfsema, Lamme, & Spekreijse, 1998; Zhou, Friedman, & von der Heydt, 2000; Roelfsema, Tolboom, & Khayat, 2007; Gilbert & Li, 2013), to the extent that they may incorporate specific aspects of natural scenes (Felsen, Touryan, Han, & Dan, 2005; Pecka, Han, Sader, & Mrsic-Flogel, 2014; Coen-Cagli, Kohn, & Schwartz, 2015; Hesse & Tsao, 2016). Similarly, visual percepts are influenced by preceding stimuli to produce strong phenomenological experiences (Blakemore & Campbell, 1969), such as aftereffects (Anstis, Verstraten, & Mather, 1998). Perceptual judgments of elementary image elements are impacted by global characteristics of the surrounding image (Schwartz, Hsu, & Dayan, 2007; Choung, Bornet, Doerig, & Herzog, 2021), often incorporating higher-level inferences about object structure (Bar, 2004; Neri, 2014; Neri, 2017).

It is now evident that visual sensors adapt to the wider context within which they are embedded, such as the highly structured nature of environmental signals (Simoncelli & Olshausen, 2001). However, existing measurements are largely restricted to constrained viewing conditions in which human observers cannot display the full repertoire of natural vision. For example, our current understanding of large-scale contextual interactions for visual processing of image features is limited to settings in which humans view flat images on a computer monitor, with their heads immobilized and their eyes fixed (Choung et al., 2021; Neri, 2017). The conditions of these experiments present the advantage of fine experimental control (Rust & Movshon, 2005), but they do not adequately capture natural vision in terms of stimulus richness and ecological validity of human behavior (Felsen & Dan, 2005).

I overcome the above limitations by performing quantitative measurements of visual discrimination in virtual reality (VR). To my knowledge, this study is the first to characterize low-level visual mechanisms during complex exploration of ecologically valid environments. VR affords the opportunity to define structural consistency of the stimulus on a much more complex level than available with traditional laboratory methods (Warren, Rothman, Schnapp, & Ericson, 2017). For example, we can define a statistical construct that specifies the ecological validity of shadow casting: whether it is consistent with a natural light source that behaves like the sun (Mamassian, Knill, & Kersten, 1998) and/or whether it aligns with active goal-oriented exploration of the environment (Gibson, 1979). VR allows fine control over complex constructs of this kind (de Gelder, Tsyri, & de Borst, 2018).

Surprisingly, I find that the long-term statistical reliability of such global environmental constructs is able to steer local processes that analyze visual signals on the subsecond scale. More specifically, local edge information is anchored more effectively to objects when shadows are statistically reliable. When shadow information is unreliable, observers shift their perceptual weight toward image-based cues to the detriment of object-based cues. Dynamic reallocation of cue information happens slowly, exposing the operation of a temporally extended adaptive mechanism that gathers environmental data over timescales of minutes. Overall, the results indicate that the concept of sensory adaptation should be expanded and reframed to incorporate not only the proximal spatiotemporal context of sensory stimuli, but also the full perceptual experience of agents engaged in active exploration of complex environments (Gibson, 1979).

## Methods

### VR hardware and software

I collected data with two head-mounted devices (HMDs): HTC Vive and HTC Vive Pro Eye. The latter supports eye-tracking capabilities. The nominal field of view provided by the manufacturer is 110 degrees (horizontal), but the actual field of view is known to be much smaller (∼65 degrees) under typical viewing conditions (Lange, Stratmann, Gruenefeld, & Boll, 2020). In the conditions of the experiments, I estimated a usable (relatively undistorted) horizontal field of view of ∼60 degrees. Two tracking cameras (HTC Vive Base Stations) were placed on opposite sides of the physical test area (measuring approximately 3 × 3 m) at a height of ∼2 m. The HMD was cabled to an Alienware Area 51 desktop and directly controlled from Unity. All virtual elements, shaders, active routines, and image-processing modules were supported by in-house software written in C#. Participants were monitored at all times during experimentation to ensure safe navigation.

### Participants and data mass (number of trials)

All protocols received ethics approval (agreement RIPH3 #2022-A00963-40), and all participants gave informed consent. I tested 10 naive participants and 1 non naive participant (author PN). Each participant collected between 600 and 1,200 trials, equally split across the three shadow configurations, where “trial” refers to one sensory discrimination event. I collected a total of 9,300 trials. Different shadow configurations were run in different blocks of 100 trials in pseudo-random order. Participants were paid 15 euros per hour.

### Virtual room design

**Figure 1.**
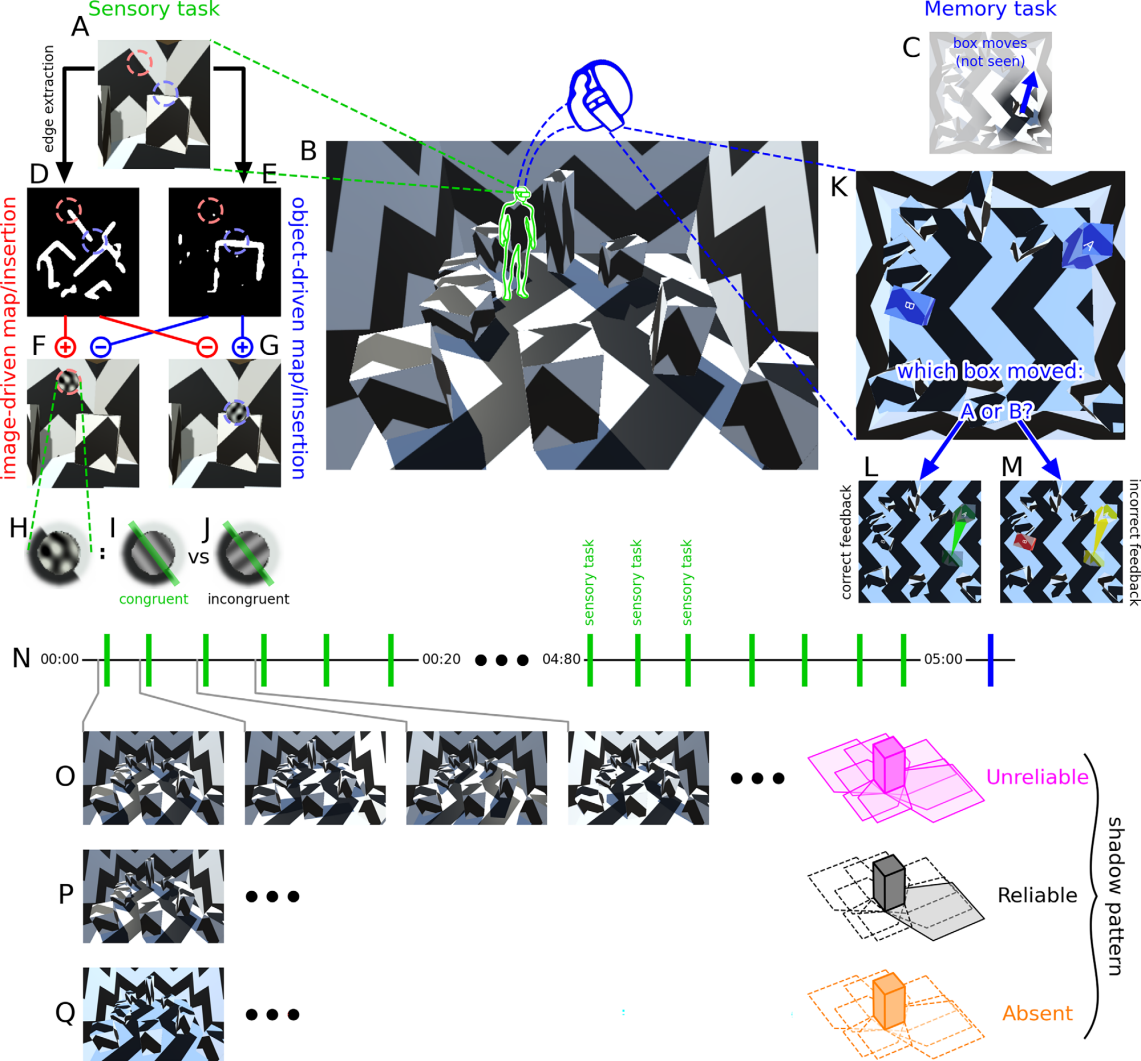
Sensory/memory tasks in an edge-rich VR world. Observers operate within a virtual room containing numerous edges (**B**). Every few seconds, the scene viewed by observers (**A**) is analyzed to extract an image-driven map (**D**), reflecting edge-contrast energy in the 2D scene, and an object-driven map (**E**), reflecting object edges defined by 3D layout (see Methods). Guided by these maps, oriented probes (circled in **F**–**G**) are locally inserted into either image-defined edges (**F**, red) or object-defined edges (**G**, blue) and corrupted by orientation noise (**H**). In the sensory task, observers must determine whether probe orientation is congruent (**I**) or incongruent (**J**) with the orientation locally defined by the scene (indicated by green line in **I**–**J**). After 100 such discriminations (indicated by green segments in **N**), observers are teleported above the room (blue head icon in **B**) and asked to perform a memory task (indicated by blue segment in **N**): They must identify which of two boxes moved during teleportation (**C**, **K**) and are provided with immediate feedback (correct/incorrect, **L**/**M**). In different experiments, cast shadows are structured according to three configurations: “reliable” (**P**), in which shadows are consistent with one fixed source of illumination throughout the 5-min block; “absent” (**Q**), in which shadows are completely removed from the environment; and “unreliable” (**O**), in which shadows are recast to conform with a different source of illumination half a second before the presentation of each probe (events indicated by connecting lines between **N** and **O**).

The rendered dimensions of the virtual room were 6 × 6 × 3 m (width × length × height). The interior of all sides (including floor and ceiling) was lined with zigzag patterns of alternating black and white stripes. Each stripe was ∼70 cm wide and changed orientation by 90 degrees every ∼1.5 m (see pattern in Figures 1B, K). I selected zigzag patterns from a wider range of high-contrast patterns, which I tested during the piloting stages of this project. Some other patterns, such as thresholded low-pass two-dimensional (2D) noise, carried the advantage of more natural appearance, but they posed serious challenges for the probe insertion algorithm, because they often lacked clear edges and did not support the corner exclusion strategy adopted by the algorithm (see below). The piloting results indicated that zigzag patterns were the only feasible option (among those tested) that would ensure successful probe insertion on a sufficient number of trials for viable experimentation. The room was filled with 10 boxes, created and specified sequentially according to the following rules. For each box, the computer algorithm selected a random location within the region defined by the floor, such that the box was not located within 80 cm from the center of the room, and its distance from all other boxes was at least 80 cm. The algorithm then selected four values for width, length, height, and rotation around the vertical axis by randomly sampling from uniform distributions over the following ranges: 40–120 cm (width), 40–120 cm (length), 40–240 cm (height), and 0–180 degrees, respectively. These rules ensured sufficient separation between boxes and avoided instances in which participants (who always started at the center of the room) would find themselves inside a box at the start of the block. The algorithm created specifications for 11 boxes but only instantiated 10 boxes during the experimental block. At the end of each block, one of the 10 instantiated boxes was moved to the location specified by the 11th box, without modifying its size and rotation (see section below on memory task). All sides of all boxes were lined with a zigzag pattern similar to the one applied to the room, except scaled down by a factor of 2.

### Lighting

When present, the point light source was located at an elevation of 45 degrees and a randomly selected azimuth value spanning the entire directional range (0–360 degrees). In the reliable shadow configuration (Figure 1P), the light source was created once at the beginning of each block and remained fixed throughout the block (it was only removed after teleportation). In the unreliable shadow configuration (Figure 1O), 500 ms before each probe appearance, the light source was destroyed and immediately re-created with a different azimuth value. In the absent shadow configuration (Figure 1Q), the light source was never created, and ambient intensity was doubled to compensate for the associated loss in general lighting. More specifically, ambient luminance in the reliable/unreliable shadow configurations ranged between ∼20 cd/m^2^ and ∼90 cd/m^2^ with an average of ∼60 cd/m^2^, depending on which part of the room was viewed at the time of measurement. I obtained these estimates indirectly by placing a photometer against one the two lenses inside the HMD and by sampling the environment for different HMD directions/positions similar to those adopted by observers during the experiments. These figures are therefore only indicative of the broad luminance range spanned by the stimuli and should not be regarded as accurate measures. Based on similar measurements in the absent shadow configuration, I determined that it was necessary to increase ambient intensity for this configuration to match the other two shadow configurations. This match could only be achieved in terms of average luminance intensity, not in terms of range: Reliable/unreliable shadow configurations inevitably spanned a larger range compared with the absent shadow configuration.

### Sensory task

#### General procedure

Observers were asked to judge the orientation of a visual probe that was briefly flashed every few seconds. The probe lasted 200 ms, and different probe presentations were separated by random time intervals uniformly distributed between 2.0 and 3.6 s. Immediately after probe disappearance, observers were given 1 s to produce a valid binary response by pressing one of two buttons on the handheld HTC Vive Controller. Their response choice was between “congruent” and “incongruent” (defined further below). At the end of the 1-s response interval, they were provided with visual feedback indicating whether their response was correct, incorrect, or invalid (no response within the 1-s window), and how many consecutive incorrect/invalid responses they had produced. See below for further details on probe construction, feedback delivery, and rules associated with correct/incorrect responses.

#### Probe object construction

Although the probe was inserted into the virtual environment, which was defined in three dimensions, it was essentially a 2D object: It consisted of a disc with zero thickness and no identifiable depth. In this sense, the probe lived in retinal space. This dissociation between the three-dimensional (3D) environment and the 2D nature of the probe was achieved via the following specifications. First, the disc element supporting the probe was always placed at a fixed distance from the observer (50 cm), and always oriented in 3D space to face the observer: When viewed from the HMD, the probe always appeared as a disc of constant diameter defined in visual space, spanning ∼3 degrees of visual angle. Second, the disc was not subjected to shadow rendering and was only illuminated by a designated light source placed at the center of the HMD, ensuring that the luminance pattern within the probe was under direct control of the texture pattern applied to the disc. More specifically, the disc was not illuminated by the light source that illuminated the room, and the room was not illuminated by the light source that illuminated the disc. Third, the disc was presented monocularly to a randomly selected eye, accompanied by a uniform gray disc of matched average luminance presented to the other eye. I adopted a slightly larger diameter for the uniform disc (∼3.3 degrees) to accommodate potential misalignments associated with vergence. Under this dichoptic configuration, informal assessment by all participants indicated that the probe was clearly visible and did not engage in binocular rivalry with the uniform disc. Furthermore, it did not produce a percept of stereoscopic depth.

#### Probe texture specification

The texture applied to the probe consisted of 16 superimposed gratings, windowed by a disc with a Gaussian edge profile of SD ∼15 arcmin (Figure 1H). Probe design conformed to extensively validated specifications that yielded meaningful results in prior applications outside VR (Neri, 2014; Neri, 2017). Each probe was defined by two vectors, each containing 16 elements: an amplitude vector **a** and a phase vector **p**. Different elements *a*_*i*_ of **a** specified the different amplitude values of an array of 2D gratings spanning the entire orientation axis in 16 equally spaced steps. A similar convention applied to **p**. For example, *a*_1_ and *p*_1_ specified the amplitude and phase of a grating oriented at 0 degrees, *a*_2_ and *p*_2_ specified the amplitude and phase of a grating oriented at 11.25 degrees, *a*_3_ and *p*_3_ specified the amplitude and phase of a grating oriented at 22.5 degrees, and so on in steps of 11.25 degrees until *a*_16_ and *p*_16_ specified the amplitude and phase of a grating oriented at 168.75 degrees. Frequency was fixed at ∼1.2 cycles/deg. The probe simply consisted of the sum of all gratings specified by **a** and **p**. Phase was randomly drawn from a uniform distribution between 0 and 360 degrees for each element of **p**. The amplitude vector **a** was obtained by summing two amplitude vectors **n** and **s**. Each element of the noise vector **n** was drawn from a Gaussian distribution typically centered on 3% contrast with SD of 1% contrast, although these exact values were adjusted individually to target threshold performance (d′ ∼1). Each element of the signal vector **s** was set to 0, except for the two elements associated with gratings oriented at 0 degrees and 90 degrees: Either one was assigned a value of 40% contrast (the other element was set to 0). When the probe was rendered, the 16 orientation values associated with the above specification were shifted by an amount equivalent to the orientation specified locally by the environment (see below for further details on how this characteristic was determined): 0 degrees above corresponds to the orientation specified by the environment, which I term “congruent,” and 90 degrees corresponds to the orientation orthogonal to the orientation specified by the environment, which I term “incongruent.” When rendered, average probe luminance was ∼50 cd/m^2^ as estimated via indirect photometric measurements (see above).

#### Probe insertion algorithm

The overall orientation of the probe was specified by the local orientation defined by the environment at the location where the probe was inserted. I refer to said location with the term “insertion point.” Right before probe presentation, two maps of the environment were captured from the HMD: an image-driven map, consisting of a 2D snapshot of the scene viewed by the observer, taken by a cyclopean camera positioned between the two eyes, and an object-driven map, consisting of a depth map of the scene where the intensity of a given pixel scaled with the distance of the corresponding environmental location from the observer. Based on calibration tests with fiducial markers placed at the corners of the captured area, I estimate this area to cover 45 × 50 degrees (width × height) of the central visual field. The image-driven map was converted to grayscale, so that the two maps were both defined by 2D matrices of matched dimensions and aligned coordinates in visual space. They were downsampled to 90 × 100 pixels (width × height) to achieve rapid image processing, which was implemented primarily using the OpenCV for Unity package (C# port). Each map was subjected to edge detection via Sobel filtering. The resulting image-driven and object-driven edge maps (Figures 1D–E) were then used to identify two potential insertion points: an image-driven insertion point (red dashed circles) and an object-driven insertion point (blue dashed circles). When describing the identification of an image-driven insertion point below, I refer to the image-driven edge map as “driver” map *M*_↑_ and to the object-driven edge map as “modulator” map *M*_↓_. The role of the “driver” map is to direct the probe insertion point *toward* the information content represented by said map (image-based if the driver map is defined by the image-driven edge map, object-based if the driver map is defined by the object-driven edge map). The role of the “modulator” map is to direct the probe insertion point *away* from the information content represented by said map. Identification of an object-driven insertion point simply involves swapping denominations for the two maps: The object-driven edge map becomes “driver” (*M*_↑_), and the image-driven edge map becomes modulator (*M*_↓_). Both maps were initially normalized to range between 0 (minimum value within map) and 1 (maximum value within map). *M*_↓_ was blurred with a Gaussian kernel of SD equal to 5 pixels and sign-inverted as *M*_↓_ → 1 − *M*_↓_ (0 corresponds to maximum value within original map, and 1 corresponds to minimum value within original map). Each entry of *M*_↑_ was corrupted by a noise source uniformly distributed between 0 and 0.2 to introduce a small degree of variability in the insertion point for a given view. The algorithm then set corner regions within *M*_↑_ to zero. These regions were identified by subjecting *M*_↑_ to Harris corner detection with block size 10, aperture parameter 3, and Harris detector-free parameter 0.04, to obtain a corner map *M*_⌟_. This map was thresholded to retain only the top 10% of its values, which were set to 0 while all remaining values were set to 1, and *M*_↑_ → *M*_↑_ × *M*_⌟_. The algorithm then combined *M*_↑_ and *M*_↓_ to obtain the insertion map *M* = *M*_↑_ × *M*_↓_. *M* was windowed by a circular envelope to include only the central disc of diameter equal to 80% of image width, with a tapering Gaussian edge of SD equal to 10% of image width. The final probe insertion point corresponded to the pixel with maximum intensity within *M*. Local orientation was defined as the orientation returned by the Sobel detector for that location within *M*_↑_. On each trial, the decision as to whether the probe would be inserted at the image-driven insertion point or at the object-driven insertion point was taken randomly with equal probability, meaning that (on average) the number of trials containing image-driven insertions matched the number of trials containing object-driven insertions.

#### Online correction of probe insertion point

I estimated that probe insertion involved a delay of ∼60 ms with respect to the time at which image information was acquired for determining the insertion point (see above). During this time, observers could move their head to face a slightly different scene, causing potential mismatches between intended and actual insertions. To compensate for this spatial misalignment, the correction algorithm calculated the difference in HMD direction between the time of image acquisition and probe insertion, and applied an equivalent shift of spatial coordinates to the nominal insertion point. For example, if observers moved/rotated their head to the right, this would cause a leftward shift of the intended insertion point in retinal coordinates. The correction algorithm therefore modified the horizontal coordinate returned by the insertion algorithm described above to reflect this shift in retinal image. Based on qualitative evaluation of the insertion algorithm while wearing the HMD, this procedure improved the accuracy of the insertion algorithm. However, it did not completely eliminate all misalignments, which occasionally occurred particularly in the case of fast HMD movements.

#### Feedback element

Feedback was delivered by a colored disc presented at fixation for 140 ms, using the same 2D support implementation techniques adopted for the probe (HMD-facing orientation, independent illumination, monocular presentation). The feedback disc had a fixed diameter of ∼9 degrees and opacity of 50%. It appeared at the end of the response period (see above) and was solid green when preceded by a correct response, solid red when preceded by an incorrect response, and open red when preceded by an invalid response. On incorrect trials, the red disc included a numeric digit placed at its center, which indicated the number of consecutive incorrect trials at that point throughout the block. This number was reset to 0 every time observers produced a correct response. Observers were provided with real-time information about the number of consecutive incorrect trials because this parameter determined the occurrence of a highly disruptive event with important consequences for their final performance score (see below).

The potential role of feedback in determining the strategy adopted by participants was not specifically explored in this study. Feedback was provided to ensure that the above monitoring procedure could be implemented, facilitate familiarization with task requirements, and prompt observers to maximize their sensory performance. It is possible that it also played a role in steering participants toward adopting strategies that, had feedback been withheld, they may not have otherwise adopted. However, it is unlikely that this phenomenon would be specifically connected with the image-based/object-based distinction, because participants were not aware of this distinction in relation to how the insertion algorithm operated. When feedback was incorrect on trials for which they were confident that their response was correct, they never reported that they explicitly ascribed this inconsistency to the probe insertion algorithm. Furthermore, any potential impact of feedback is expected to remain unchanged across shadow configurations, thus mitigating the importance of this issue for the present investigation. At the same time, the potential role of feedback remains an interesting and important topic for further inquiry in future work.

### Memory task

The memory task was introduced to engage observers in active encoding of scene layout. After 100 presentations of the sensory probe (approximately 5 min), position tracking was disabled and the HMD was teleported to a virtual location above the room that corresponded to the observer’s feet standing on a transparent ceiling. Rotation tracking was left active. From this position, observers could inspect the room immediately below their vantage point by rotating their head (and moving their eyes), but they could not navigate laterally. Cast shadows were absent in this configuration, regardless of which shadow configuration was adopted during the preceding block. During teleportation, one of the 10 boxes in the room (randomly selected) was moved to the 11th box location (which had not been previously occupied by any box), without changing its size and rotation. This event was not seen by the observer. The displaced box and one other randomly selected box were highlighted by applying a blue tint to their texture, and were randomly labeled with the letters “A” and “B” (see Figure 1K). Observers were asked to identify the displaced box by pressing one of two buttons on the controller (one button for A, the other button for B). Immediately after responding, feedback was provided in the form of duplicating the displaced box at its original position and via application of specific color changes to the boxes. The duplicate box was semi-transparent to distinguish it from the displaced box and to emphasize its belonging to a past event. In the event of a correct response, a green tint was applied to the displaced box and to its duplicate. The two boxes were connected by a green element indicating the direction of displacement in the form of an expanding strip (Figure 1L). In the event of an incorrect response, a red tint was applied to the selected box, and a yellow tint was applied to the displaced box, to its duplicate, and to their connecting element (Figure 1M). Observers were introduced to both sensory and memory tasks during brief training sessions preceding data collection. They were therefore aware that they would be required to perform the memory task at the end of each block.

### Rules for preventing single-task strategies

I introduced a set of rules specifically designed to ensure that observers would perform both sensory and memory tasks while inhabiting the virtual room. This design feature is important, because failure to perform either task would significantly impact the behavior intended for the sensory task, which is the task of primary interest for the measurements adopted here. More specifically, if participants were to focus on the memory task while ignoring the sensory task, performance in the latter task would drop to chance. Conversely, if participants were to focus on the sensory task while ignoring the memory task, the most productive strategy for performing the latter task involves staring at a fixed region of the room, possibly a wall, to reduce uncertainty about probe appearance and facilitate sensory performance. This scenario would not be representative of natural vision and would produce redundant insertions with poor probing power, making any associated results difficult to interpret in meaningful ways. I therefore wished to avoid both scenarios and prompted observers to perform both tasks at the same time.

The experiment was presented to observers in the form of a game with a point-based score. To ensure that observers would not be able to ignore the sensory task, I introduced the following rules for point assignment. Each correct sensory discrimination counted 1 point. At the end of the block, their total score was doubled if they responded correctly in the memory task or halved if they responded incorrectly. After four consecutive incorrect/invalid responses, all boxes within the room disappeared and immediately reappeared in a completely new configuration (using the algorithm for box placement/sizing/rotation detailed above). I refer to this event as “room scrambling.”

The above rules undermine single-task strategies for the following reasons. If observers only focus on the memory task while ignoring the sensory task, their drop in sensory performance will result in fewer points at the end of the block. If they choose to rely on the residual correct responses generated by chance in the sensory task, they nevertheless run into the risk of producing several consecutive incorrect responses, with the resulting room scrambling event nullifying their efforts to memorize room layout up to that point. Conversely, if observers only focus on the sensory task while ignoring the memory task, they risk halving their score (and missing out on potentially doubling it) at the end of the block. Observers clearly understood these implications during data collection. As a consequence, they never ignored either task.

### Memory task without sensory task

In additional experiments, I removed all probe presentations and instructed observers to perform only the memory task. Under these conditions, I found that the 5-min block duration adopted in the dual-task experiments resulted in ceiling performance for the memory task (100% correct responses). To target threshold performance comparable with the levels achieved in the dual task (∼75% correct responses), I reduced block duration to 30 s. Except for removing the sensory probes, all other experimental manipulations were left unmodified.

### Estimation of sensitivity and response bias

I estimated sensitivity and response bias from valid trials (I excluded trials with invalid responses) using the two metrics d′ and criterion *c*, computed using standard formula from signal detection theory (SDT; Green & Swets, 1966): d′=$\Phi^{-1}\left(p_{\mathrm{hit}}\right)-\Phi^{-1}\left(p_{\mathrm{fa}}\right)$ and *c* = $-[\Phi^{-1}\left(p_{\mathrm{hit}}\right)+\Phi^{-1}\left(p_{\mathrm{fa}}\right)]/2$, where $p_{\mathrm{hit}}$ is the hit probability, $p_{\mathrm{fa}}$ is the false alarm probability, and Φ^−1^ is the inverse of the normal cumulative distribution function. Our goal is to determine whether the sensory process probed by the measurements is strictly visual or decisional. I formulate these two positions in the following (deliberately accentuated) terms: By “strictly visual,” I mean that the sensory mechanisms underlying edge detection/representation undergo measurable alterations of their response characteristics, such as changes in their orientation tuning and/or selectivity for spatial frequency; by “decisional,” I refer to changes in the criterion adopted by observers to produce a binary response. The latter possibility must be explicitly entertained because the adopted task conforms to the yes/no protocol (Green & Swets, 1966), and the two possible responses (congruent vs. incongruent) are not symmetric: Under these conditions, it is entirely reasonable to expect response bias.

To arbitrate between the two scenarios outlined above, behavioral scientists typically estimate two independent quantities (Green & Swets, 1966): changes in d′, which are meant to capture genuine changes in the ability of the underlying sensory mechanism to extract signal-relevant information from the stimulus, and changes in criterion *c*, which are meant to reflect changes in the disposition of the participant toward more lax (inclined toward reporting congruent) or more conservative behavior (inclined toward reporting incongruent). Estimation of these two quantities from binary responses typically involves committing to the equal-variance SDT model. Because the equal-variance assumption is likely violated under many real-world scenarios (Green & Swets, 1966), the resulting d′ and *c* estimates reflect mixtures of sensory and decisional effects. In other words, these metrics cannot be expected to achieve full orthogonalization of the cognitive mechanisms they are meant to capture independently.

**Figure 2.**
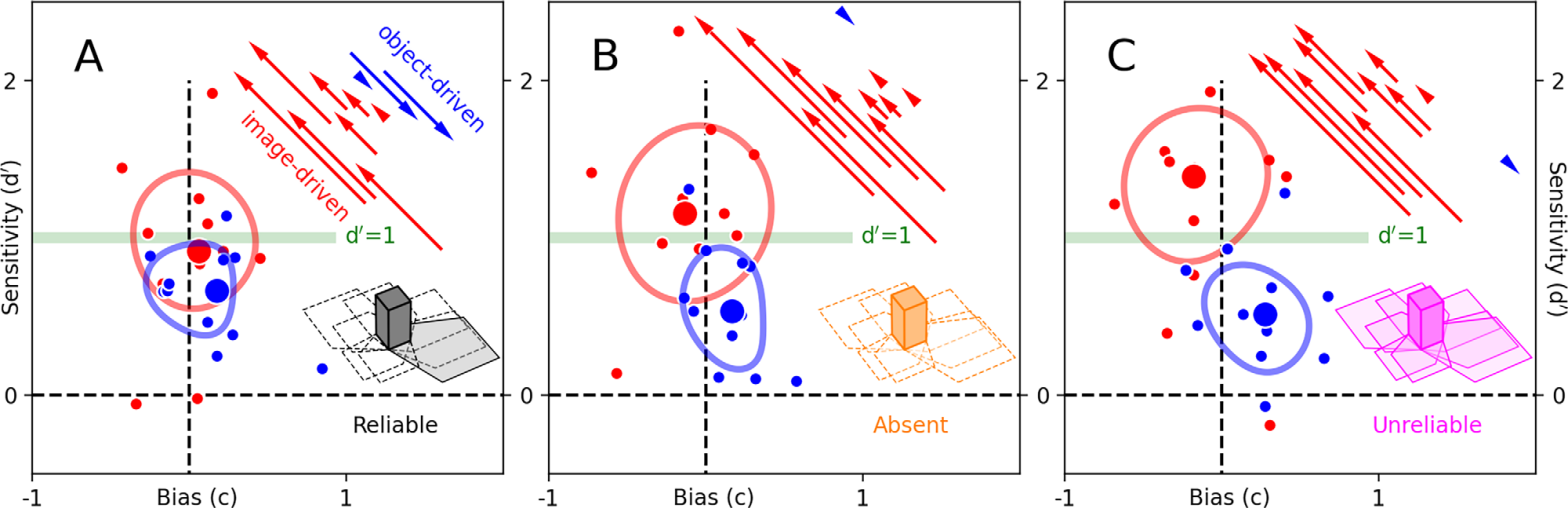
Sensitivity shifts in favor of image-driven information when shadow information is absent/unreliable. When shadow casting is reliable (**A**), sensitivity (y-axis) and response bias (x-axis) for performing the sensory task (congruent/incongruent orientation discrimination; see Figures 1A–J) are similar between image-driven (red symbols) and object-driven (blue symbols) probe insertions: Data points scatter around a sensitivity (d′) value of 1 (indicated by horizontal green line) and no response bias (0 on the x-axis, indicated by vertical dashed line). Diagonal arrows (top-right region of the plot) connect object-driven (arrow base) and image-driven (arrow end) projections of sensitivity/bias values onto the negative diagonal for each observer. Arrow color is red if image-driven projected value falls to the left of object-driven projected value, blue otherwise. When shadow casting is absent/unreliable (**B**–**C**), image-driven and object-driven data clusters shift away from each other along the negative diagonal. This shift may be informally characterized as a perceptual “flattening” effect (see main text). Contours reflect data spread using an automated smoothing/thresholding procedure and are intended for visualization only (see main text for statistical analysis).

To assess/address the crossover between sensitivity and response bias at the level of d′ and *c* metric estimates, and determine whether the main effects are attributable to one or the other cognitive mechanism, I evaluated the size and consistency of the two metrics. As detailed in the Supplementary Text and shown in Supplementary Figure S1, the data decisively support the conclusion that the cluster separation effects in Figure 2 are driven by sensitivity changes and not changes in response bias. More specifically, I find that *c* estimates produce small and inconsistent effects, thus likely reflecting estimation errors deriving from imperfect separation of sensitivity and response bias.

### Modeling

The proposed model should not be intended as a mechanistic explanatory account of the underlying cognitive processes. It is best intended as an illustrative tool for clarifying my interpretation of the results, which is based on cue reweighting (Hillis, Watt, Landy, & Banks, 2004). In this sense, the proposed implementation of the early visual processing stages (e.g., edge detection, object identification) is neither veridical nor exclusive, and the proposed model does not carry much explanatory power beyond the specific conditions of the experiments reported here.

#### Stimulus/response information fed to the model

The model was provided with the following data for every experimental trial (I excluded trials with invalid responses): a monochrome (average intensity across color guns) image-based snapshot *M*_*image*_ of the scene right before probe insertion (Figure 4A), a depth-based snapshot *M*_*depth*_ of the scene right before probe insertion (not shown), an image-based snapshot *M*_*probe*_ of the scene during probe insertion (Figure 4F), and the binary congruent/incongruent response generated by the human observer (Figure 4G). The three input images were used by the model to generate a congruent/incongruent response (Figure 4D), which was then compared against the response generated by the observer (see below).

**Figure 3.**
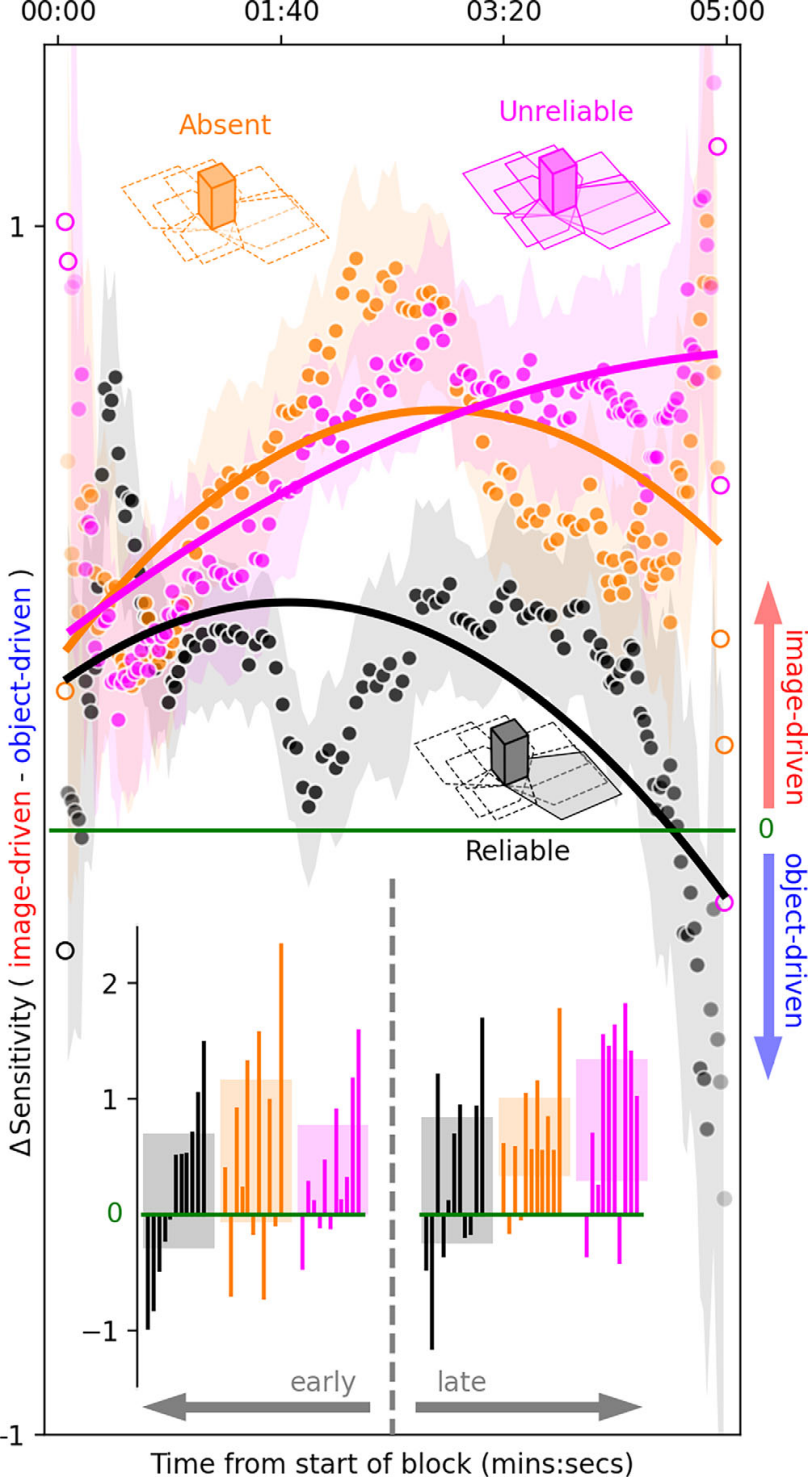
Absent/unreliable shadow configurations slowly drift away from reliable configuration. Difference in sensitivity (ΔSensitivity) between image-driven and object-driven insertions (y-axis) is plotted for different time points (x-axis) during the 5-min experimental block over a 2-min sliding window centered on each point. This quantity, which corresponds to the distance between red and blue clusters along the y-axis in Figure 2, is similar for different shadow configurations at the beginning of the block (leftmost data points). As time progresses (moving rightward along the x-axis), data for the absent/unreliable shadow configurations (orange/magenta) depart from corresponding data for the reliable-shadow configuration (black), until they diverge to significantly positive values of ΔSensitivity toward the end of the block (rightmost data points) that reflect the shifts reported in Figures 2B–C. Histograms within insets plot ΔSensitivity values for different observers (one bar per observer) in the three shadow configurations (color-coded as detailed above), computed separately from two equal subsets of data corresponding to “early” and “late” halves of the block. Shaded regions within main panel indicate ±SEM (open symbols refer to data points for which SEM could not be computed because of insufficient data); those within insets indicate 95% confidence intervals across observers. Solid curves plot univariate spline fits of degree 2 and are intended for visualization only.

**Figure 4.**
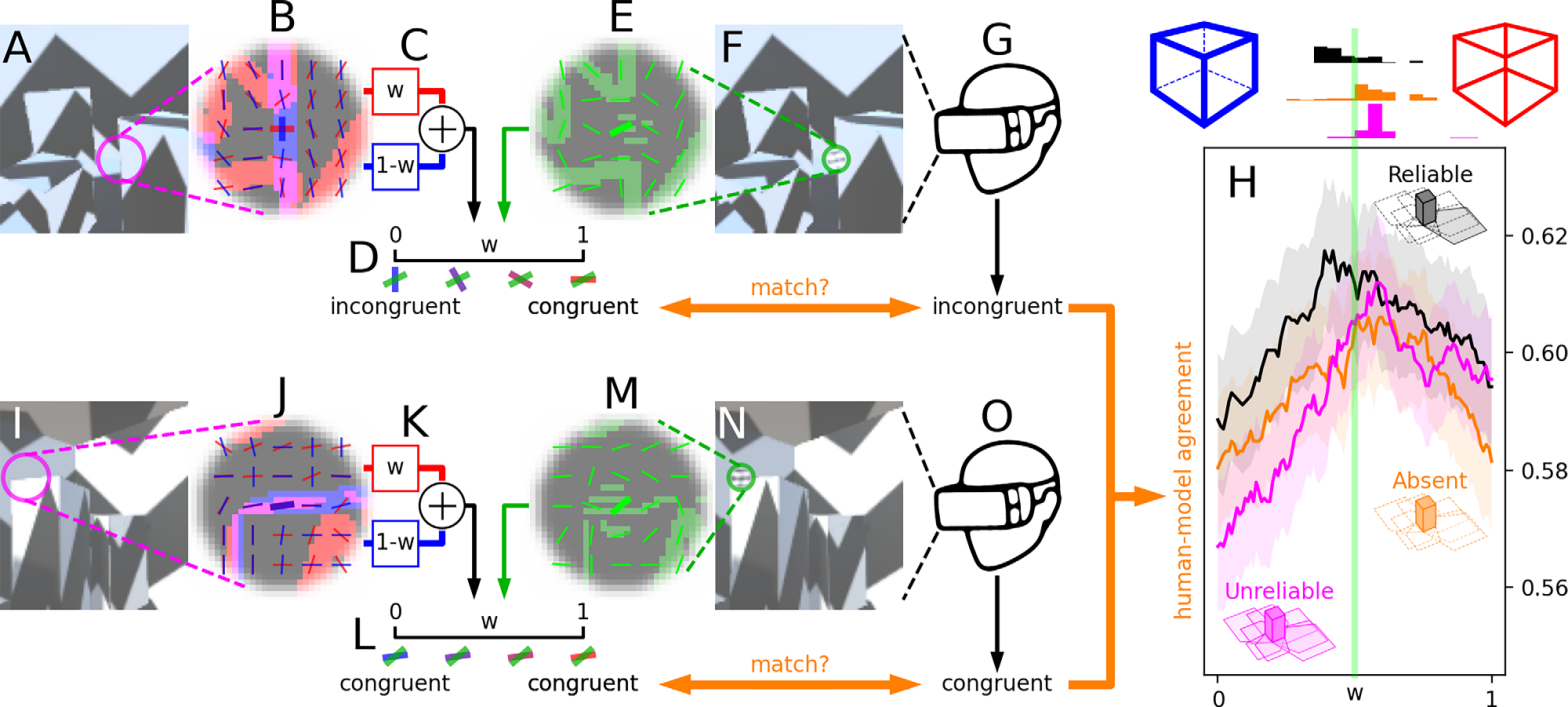
Trial-by-trial modeling of human responses via flexible cue combination. For every experimental trial, the model is provided with image information captured directly from the HMD right before (**A**) and during probe insertion (**F**). It then constructs an image-driven energy map (red regions within surface plot in **B**) with associated orientation map (red segments in **B**), and an object-driven map (blue regions) with associated orientation map (blue segments). The model then combines these two orientation maps via weight *w* (**C**): When *w* = 1, only the image-driven map is used; when *w* = 0, only the object-driven map is used. Intermediate values of *w* involve mixtures of the two maps (see Methods). Based on this weighted combination, the model estimates the overall orientation of a local region around the probe insertion point (indicated by magenta circle in **A**). Different values of *w* may correspond to different orientation estimates (blue/red segments in **D**; hue value between blue and red reflects *w* value). The model also constructs a descriptor from **F**: an image-driven energy map (green regions within surface plot in **E**) with associated orientation map (green segments in **E**). The model then uses this orientation map to estimate probe orientation from the local region occupied by the probe (indicated by green circle in **F**). This estimate is directly compared with the estimate obtained from **A** to determine whether probe orientation is congruent or incongruent, which in general will depend on the value of *w*: For this example trial, *w* = 0 corresponds to an “incongruent” response, and *w* = 1 to a “congruent” response (**D**). The same procedure is applied to the next trial, as shown in **I**–**N**. On this trial, the value of *w* does not impact the final response (**L**) because image-driven and object-driven orientation maps agree within the probe region (**J**). Model responses are matched against human responses on corresponding trials (**G**, **O**) to compute human–model agreement (fraction of matching responses) across all trials. Agreement (y-axis in **H**) reaches its maximum for different values of *w* (x-axis in **H**) when computed from different shadow configurations (compare traces of different colors). Histograms (top of **H**) plot distributions of *w* values that maximize agreement across multiple bootstrap iterations: They are mostly distributed to the left of *w* = 0.5 (this value corresponds to equal weight between image-driven and object-driven information, indicated by vertical green line) for the reliable shadow configuration (black histogram), and mostly to the right of *w* = 0.5 for absent/unreliable configurations (orange/magenta histograms). Shaded regions in **H** show ±1 SD across iterations.

#### Map construction

The image-processing strategy used for the model largely mirrored the strategy adopted for probe insertion (see above), albeit with some modifications meant to reflect known properties of primary visual cortex. The algorithm submitted *M*_*image*_ to energy extraction by a quadrature pair of Gabor wavelets with carrier frequency equal to 1 cycle per 2% of image width (equivalent to ∼1 cycle/degree when referred back to visual space), SD of Gaussian envelope equal to 2% of image width (equivalent to ∼1 degree), and oriented at one of eight values uniformly spanning the 0–180 degree range. For each pixel across the image, the algorithm selected the orientation corresponding to the largest energy output to construct an image-driven orientation map (red in Figure 4B). The algorithm repeated this process for *M*_*depth*_ and computed the corresponding object-driven orientation map (blue in Figure 4B). The algorithm also repeated this process for *M*_*probe*_ after halving the SD of the Gaussian envelope and computed the corresponding probe orientation map (green in Figure 4E). I reduced the spatial extent of the Gabor filters (smaller SD) for the latter map to reflect more localized processing of the probe, which we expect to simulate the perceptual processes engaged by observers. More specifically, we expect that, before gaining knowledge of probe location, observers would carry out relatively coarse orientation estimation across the visual field (indicated by the magenta circle in Figure 4A). When the probe appears, we expect observers to engage in more localized processing of the region around the probe (indicated by the green circle in Figure 4F), hence the smaller SD value adopted to simulate the latter process. This strategy is effectively prompted by the nature of the sensory task: Observers are essentially asked to compare the local orientation of the probe with the overall orientation of the surrounding scene, because this comparison forms the basis for the congruent/incongruent judgment.

#### Decisional stage

The algorithm computed the circular difference between the image-driven orientation map and the probe orientation map. The resulting image-driven differential map contains orientation values between 0 and 90 degrees. The algorithm also computed the corresponding object-driven differential map (circular difference between the object-driven orientation map and the probe orientation map) and combined the two differential maps via weighted averaging: Factor *w* was applied to the image-driven differential map, and factor 1 – *w* was applied to the object-driven differential map (Figure 4C). I refer to the resulting combined map as the incongruency map: It reflects the degree of incongruency (orientation distance) between scene and probe. The algorithm averaged values from this map within a 3 × 3 box centered on probe location and produced an incongruent response if the resulting value was >45 degrees, or a congruent response otherwise (<45 degrees). On some trials, the generated orientation maps were not viable because the corresponding energy maps (see above) contained no energy within the selected pooling region. These trials, which represented a sizable proportion of the data set (∼30%), were excluded from analysis. Attempts at reducing this exclusion rate were unsuccessful because they required increasing the envelope size of the local filters and/or increasing the size of the pooling box described immediately above, which in turn produced extremely noisy estimates of local orientation content that did not support sensible outputs at the decisional stage.

#### Human–model agreement

Human–model agreement is the fraction of trials on which the model generates the same response produced by human observers, over all experimental trials across all observers. An agreement value of 0.5 indicates complete decoupling between model and human responses, while an agreement value of 1.0 indicates perfect match. In general, values fell within a tight range between 0.55 and 0.65. I computed agreement separately for different shadow configurations and for different values of *w* (Figure 4H). Agreement generally peaked for intermediate values of *w*, between 0.3 and 0.7.

### Statistical analysis

**Figure 5.**
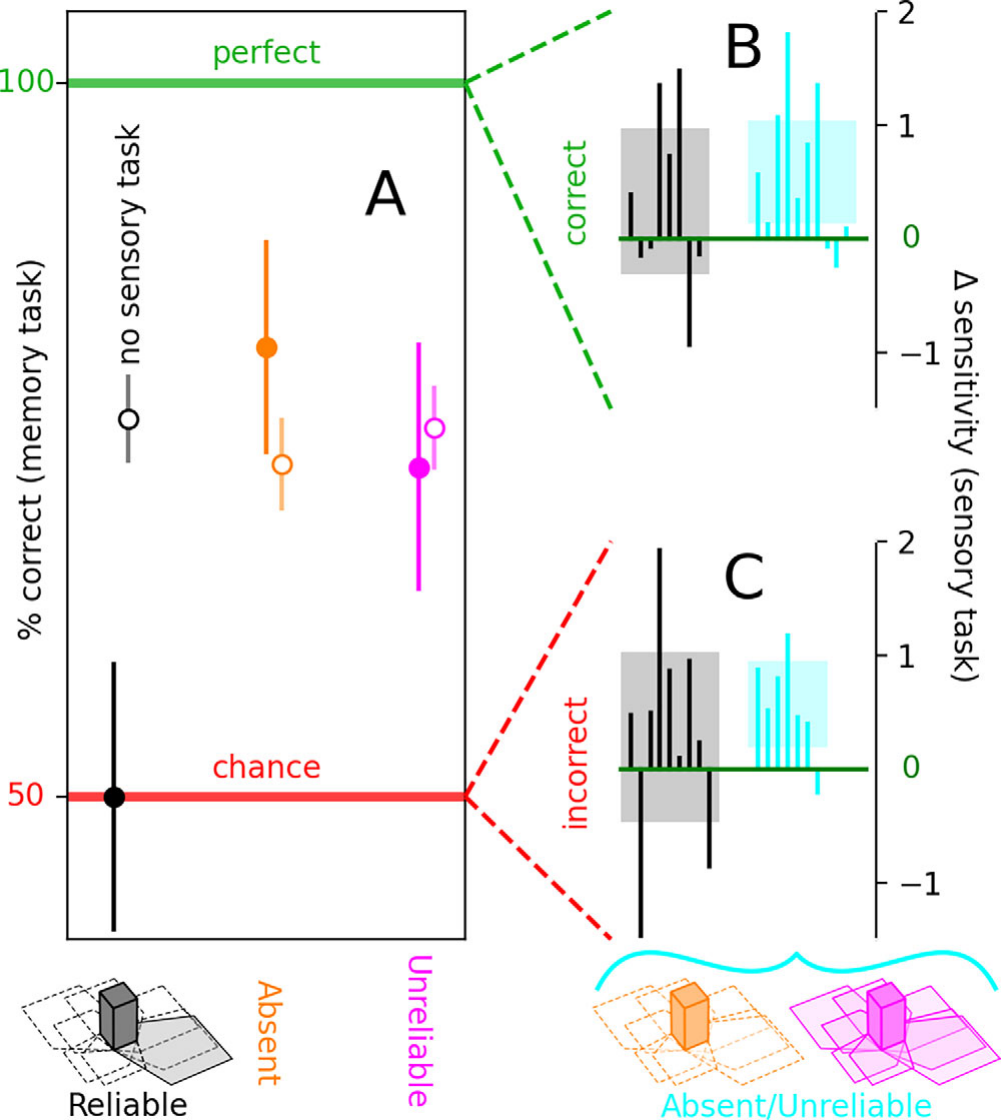
Memory performance is impacted by the sensory task, but sensory performance is not impacted by the memory task. Recall performance in the memory task (y-axis in **A**) is at threshold levels for absent/unreliable configurations (orange/magenta data points in **A**) but drops to chance for the reliable configuration (solid black data point). When the sensory task is removed (open symbols in **A**), performance is similar across all shadow configurations. Differential sensitivity in the sensory task follows similar patterns for blocks on which participants responded correctly in the memory task (**B**) and for those on which they responded incorrectly (**C**). More specifically, differential sensitivity is not different from 0 for the reliable shadow configuration (black histograms in **B**–**C**) and is positive for the absent/unreliable configurations (cyan histograms). Histograms are plotted to the conventions adopted in Figure 3.

To evaluate the statistical significance of the data pattern suggested by the visualizations in Figure 2, I followed accepted guidelines for statistical assessment of interactions (Nieuwenhuis, Forstmann, & Wagenmakers, 2011). I first carried out an exploratory two-way repeated-measures analysis of variance (ANOVA) with shadow pattern (reliable/absent/unreliable) and insertion type (image-driven/object-driven) as factors and the diagonal shift (projected values onto negative diagonal returned by *d*′−*c*) as the dependent variable. This analysis confirmed the presence of an interaction between factors (*p* = 0.03), as suggested by visual inspection of Figure 2 (I also found an effect of insertion type [*p* = 0.0034] but no effect of shadow pattern [*p* = 0.3], again consistent with the pattern exposed by the visualization). To identify which conditions produced measurable shifts, I then carried out Wilcoxon tests on diagonal shifts between image-driven and object-driven measurements, separately for each shadow configuration. I applied a 3× Bonferroni correction to compensate for the comparison across shadow configurations. The experiments were designed so that the null hypothesis adopted for the Wilcoxon tests would be transparently and unambiguously defined as involving no difference between two measurements of the same variable under two different conditions. When reporting data across individual observers for subset analysis (insets to Figure 3 and Figures 5B–C), relevant figures display 95% confidence intervals across observers in the form of shaded regions. I adopt a combination of confidence intervals and *p*-values to avoid the limitations associated with *p*-values alone (Cumming, 2014; Wasserstein & Lazar, 2016).

## Results

### Brief overview

Before unpacking the details of my measurements and their implications, I summarize their most important features here. Sensitivity for discriminating the local orientation of a brief probe embedded in a virtual environment (Figures 1F–G) is plotted on the y-axes of Figure 2. When object shadows in the virtual environment are stable, sensitivity to image-based information is comparable with sensitivity to object-based information: Red data points largely overlap with blue data points in Figure 2A. When shadows are removed (Figure 1Q) or rendered unreliable by an erratic light source (Figure 1O), sensitivity to image-based information is higher than sensitivity to object-based information: Red data points scatter above blue data points in Figures 2B–C. This difference in sensitivity, plotted on the y-axis of Figure 3, emerges slowly (timescale of minutes plotted on the x-axis) and only for the absent/unreliable shadow configurations (orange/magenta elements), not for the reliable shadow configuration (black elements). These results are successfully captured by a model that allocates more weight to image-based cues versus object-based cues when shadow information is degraded (Figure 4). Overall, these results demonstrate that slow and global characteristics of the environment (shadow distribution/reliability) can impact the operation of fast and local sensory processes (orientation discrimination of transient localized target).

### Image-based versus object-based cues for visual discrimination

Participants operated within a virtual room containing different boxes at random locations. This environment was designed to present numerous and varied edges, alongside a method to quantify whether each edge is generated by “image-based” or “object-based” elements. For example, a high-contrast edge lining the wallpaper (region indicated by red dashed circle in Figure 1A) is image-based but not object-based; conversely, the transition between a box and its background (region indicated by blue dashed circle in Figure 1A) may carry no image-based contrast but marks an object-based edge. I locally perturbed edge regions via the insertion of Gabor-like probes (Figures 1F–H) and asked observers to discriminate probe orientation (“sensory task”). More specifically, observers were asked to determine whether probe orientation was congruent (aligned) or incongruent (orthogonal) with the orientation locally specified by the environment (Figures 1I–J). Probes were selectively inserted at either image-based or object-based edges. When they were inserted along an image-based edge (region indicated by red dashed circle in Figure 1F), the congruent orientation was specified by image-based information in the environment (Figure 1D). Similarly, when probes were inserted along object-based edges (region indicated by blue dashed circle in Figure 1G), the congruent orientation was specified by object-based environmental information (Figure 1E). Probes appeared briefly every few seconds (probe events are indicated by green vertical segments along the timeline in Figure 1N). Over a period of approximately 5 min, observers therefore performed 100 instances of the sensory task. I refer to each instance as one “trial” and to the 5-min period as a “block.”

While performing the sensory task, observers were also simultaneously engaged in a separate “memory task.” In the memory task, observers were asked to memorize the layout of the room for the purpose of answering a recall question at the end of each block. When the block finished, observers were teleported above the room (blue elements in Figure 1B) so that they could view the space they previously occupied from a different vantage point (as seen in Figure 1K). Their end location was such that they were standing on the ceiling, which was rendered transparent (see Methods for details). During teleportation, one box in the room was displaced to a different location (Figure 1C). Observers were informed that this event would take place, but they did not know which box would be displaced. As they looked down onto the room, 2 of the 10 boxes were highlighted and labeled with the letters A and B (blue-tinted boxes in Figure 1K). One of the labeled boxes had been moved (box labeled A in the example shown in Figure 1K), while the other one was a randomly selected box (box labeled B). Observers were asked to indicate which, of those two boxes, had been displaced. The memory task was introduced to engage observers in explicit reconstruction of environmental 3D layout (this process is not necessarily engaged by the sensory task). Observers were therefore simultaneously performing two separate tasks on completely different spatiotemporal scales: the sensory task, reliant on local (retinotopic) fast information (subsecond timescale), and the memory task, reliant on global (room-wide) information from slow exploration (several seconds to minutes).

### In the presence of reliable shadows, both cues are used for discrimination

Figure 2A plots sensitivity (d′) for discriminating probe orientation (congruent vs. incongruent) on the y-axis, against response bias on the x-axis (see Methods for details on how these quantities were computed). Data points fall within optimal ranges for performing accurate behavioral measurements: Sensitivity falls around 1, and bias values scatter around 0. This is the sweet spot targeted by traditional psychophysical experiments, but it is not to be taken for granted that such conditions would be achieved in VR. Given the large number of extraneous factors that inevitably intervene in this medium, there are numerous reasons for expecting strong response bias. Furthermore, given the difficulty of performing a low-level discrimination task in a complex environment while concomitantly being challenged with a demanding high-level cognitive task (memory task), it is not at all trivial that observers would be able to achieve sensitivity levels substantially above chance. Finally, the technical procedure for inserting probes must operate on the fly (see Methods), which may lead to alignment failures and real-time glitches that may preclude effective performance of the sensory task. Overall, the data in Figure 2A demonstrate that these potential difficulties were successfully overcome by the adopted protocol.

When we compare the above performance metrics between insertions that were image-based (red data points) and those that were object-based (blue), we find that they are largely comparable (red and blue data clusters overlap in Figure 2A). This result demonstrates that observers were able to retrieve edge information from the scene with reference to both texture elements and object boundaries. Using imprecise terminology, we could rephrase this result by stating that observers viewed the environment as both “flat” and “in-depth.” These terms do not entirely capture the nature of the phenomena under study and may to some extent even misrepresent them. However, they are useful for an intuitive summary of the data, which motivates us to occasionally adopt them here.

### In the absence of shadows, the world becomes flat

I repeated the above measurements in the absence of cast shadows (Tarr, Kersten, & Bülthoff, 1998). Under these conditions, the environment is uniformly lit from within, without any point light source (see example in Figure 1Q). It remains in clear relief: Stereoscopic information is still available, in addition to pictorial cues about depth. However, there are no shadows. Phenomenologically, observers do not experience much difference with respect to the “reliable” configuration tested above (Figure 1P), in which shadows are present and consistent with a fixed sun-like source.

In the absence of shadows, sensitivity/bias for image-based versus object-based probe insertions falls within different regions of the plot in Figure 2B: Sensitivity is higher for image-based insertions (red data cluster is shifted upward compared with blue data cluster), and bias is slightly more lax (red data cluster is shifted leftward compared with blue data cluster). This upward-to-the-left shift can be quantified by projecting data coordinates onto the negative diagonal. The resulting values are indicated by red/blue arrows, which clearly demonstrate the shift associated with the object-based→image-based transition (*p* = 0.0058; the same analysis applied to Figure 2A returns *p* = 0.12; see Methods for details on statistical tests). This shift is in fact dominated by a change in sensitivity, while the concomitant shift in bias likely reflects imperfect dissociation between these two metrics (see Methods and Supplementary Figure S1 for further quantification).

When we compare Figure 2A with Figure 2B, we find that data clusters are pushed away from each other along the direction of higher sensitivity for image-based insertions and slightly lower sensitivity for object-based insertions. In other words, removing shadows prompts observers to increase their reliance on image-based cues, to the detriment of object-based cues (I formalize this concept further below with computational modeling). Adopting the intuitive terminology introduced earlier, we could summarize this result by stating that observers viewed the shadowless environment as being “flatter” (Tarr et al., 1998). Again, I emphasize that this characterization is rather imprecise, because it fails to capture a number of important points about the measurements reported in Figures 2A–B. More specifically, observers were never asked to explicitly rate the appearance of the environment in terms of its flatness/depthness. Rather, they were asked to perform a low-level visual discrimination task with reference to a specified local signal (Figures 1H–J). The resulting measurements are therefore properly visual and of the quantitative kind that is normally accessible only via well-controlled psychophysics. Because these measurements can be referred back to the complexity of the scene, however, they carry implications for interpreting the role played by the virtual environment.

### Sensitivity shift is connected with shadow reliability and not an artifact of low-level differences

It may be argued that any comparison between Figure 2A and Figure 2B is saddled with serious interpretational challenges associated with the many differences between shadow-rich and shadowless environments: When shadows are removed, the associated visual changes span a wide range of dimensions and cues, such as spatial frequency, edge density, orientation content, and potentially many others. From a low-level perspective, there are too many cues changing at the same time to allow informed statements about the origin of the shift discussed above. On the one hand, it is possible that this shift reflects an interesting phenomenon pertaining to higher-level reconstruction and interpretation of environmental layout: Our visual system must sort out edges into “structural” ones belonging to objects and “nonstructural” ones deriving from shadows or textures (Marr, 1982; Neri, 2017). The operation of this process would be impacted by shadow manipulations. On the other hand, it is equally possible that the shift is caused by any of several low-level image differences between the two environments, such as edge density (Bex, Solomon, & Dakin, 2009). Based on the data in Figures 2A–B, one cannot say for sure.

To address the above criticism, I designed an additional condition, which I term the “unreliable” shadow configuration. In this variant of the experiment, shadows are present and consistent with a single light source, but only for a period of a few seconds at a time: Every 3–4 seconds, and just before the probe is flashed, the light source is teleported to a different location around the room (Figure 1O). As a consequence, the shadow pattern projected onto the environment immediately changes to a completely new configuration: It is as if the sun jumped to a different part of the sky every few seconds.

A critical feature of the design presented above is that, over the timescale of the sensory task, there is no difference between reliable and unreliable shadow configurations: In both cases, observers experience a shadow pattern that is consistent with the natural laws of everyday lighting (compare leftmost scene in Figure 1O with scene in Figure 1P). More importantly, overall there are no low-level differences in visual stimulation between these two configurations: If one were to take a snapshot of the room under the two configurations at any given time, it would be impossible to tell them apart. On the longer timescale of several seconds, however, the reliable configuration is associated with a stable shadow pattern that is consistent with everyday experience, while the unreliable shadow configuration does not allow for a dependable representation of light projection from the sun, as this light source hops around in a fashion that cannot be experienced in the natural world.

Based on the above design considerations, we can make specific predictions about the manner in which data should scatter for image-based versus object-based probe discrimination. If the shift reported in Figure 2B is entirely caused by low-level image differences between reliable and shadowless environments, we should not observe such shift when shadows are unreliable: Under this configuration, there are no low-level image differences from the perspective of the fast local process that is engaged by the sensory task, and the experimental pattern should mirror Figure 2A. If, on the other hand, the shift in Figure 2B reflects the operation of a higher-level process that attempts reconstruction of 3D layout based on complex considerations about object placement and light casting, we may expect to measure a similar shift for unreliable shadows. Under this scenario, rendering shadows unreliable is not too dissimilar from removing them altogether: In both cases, information about shadow casting is degraded. Under this scenario, the experimental pattern should conform with the shift reported in Figure 2B.

As demonstrated in Figure 2C, the empirical measurements support the latter prediction: The image-based data cluster is shifted away from the object-based data cluster (*p* = 0.0088), similarly to Figure 2B. It appears that observers process the environment as “flatter” when shadows are unreliable, even though its visual appearance has not changed. What does change is the long-term statistical reliability of shadows. This factor is somehow incorporated by observers over time. It is then reverberated back to the sensory process that supports local discrimination of flashed targets, to the extent that the resulting effect can be measured via behavioral metrics. From whichever perspective they are viewed, whether conceptual or experimental, these effects are not trivially expected.

### Sensitivity shift is not an artifact of sudden environmental changes

Although the unreliable-shadow configuration allows for the exclusion of potential low-level artifacts associated with the absent-shadow configuration, it raises concerns with relation to other potential low-level artifacts of its own: In the unreliable-shadow configuration, observers experience a sudden environmental change preceding the appearance of the sensory probe, while they do not experience any such event in the reliable-shadow configuration. It may be argued that this event introduces an aspecific difference between the two shadow configurations that is unrelated to shadow reliability and may drive the data shift in Figure 2C. There are at least two ways of formulating this hypothesis.

First, the environmental change may act as a disruptive event that destabilizes perceptual processing, leading to an imbalance in the way sensory cues are combined by observers. Under this interpretation, any sudden disruptive change would generate the data pattern in Figure 2C, without requiring any specificity for shadow statistics. For example, one may apply a brief “earthquake” to the room and make all boxes wobble. This manipulation would not impact shadow reliability but would cause some degree of disruption. Second, the environmental change may act as a cueing event that warns observers about the upcoming appearance of the sensory probe. The resulting reduction in temporal uncertainty may underlie the shift in Figure 2C. Under this interpretation, any stimulus that is time-locked to the probe would produce the same effect, for example, an auditory cue.

We can reasonably exclude a role for the factors discussed above via reference to the results obtained from the shadowless configuration: In this configuration, the appearance of the sensory probe is not preceded by any disruptive/cueing event, yet we observe the same data shift that is observed in the unreliable-shadow configuration (Figure 2B). We conclude that the measured differences between reliable- and absent/unreliable-shadow configurations cannot be readily ascribed to the low-level differences introduced by the unreliable-shadow manipulation. At the same time, we must recognize that the similarity between the two sets of measurements obtained from absent- and unreliable-shadow configurations (Figures 2B–C) does not in itself guarantee that they reflect the same underlying cognitive mechanism: It remains possible that they originate from entirely different mechanisms, which nevertheless map to the same experimental signature. We cannot categorically exclude this possibility, but the more parsimonious interpretation appears to involve one and the same mechanism, thus making it unlikely that the factors discussed above played a role in determining the pattern observed for the unreliable-shadow configuration.

### Impact of environmental stability on low-level visual discrimination takes minutes to develop

In principle, we may expect the impact of the unreliable-shadow manipulation to appear within a few seconds of each block: After observers experience the first sudden change in sun location, they may become aware that shadows are not stable indicators of environmental layout. At the same time, further consideration raises the possibility that this impact may be delayed, for at least two important reasons. First, it is natural for observers to incorporate shadows into their representation of the environment as an automatic process, and one that they engage almost instantly as shadows are recast following sudden displacement of the light source. Therefore, even if they experience repeated disruption from the changing light source, observers will nevertheless engage in active reconstruction of room layout that takes shadows into account, simply because this is what their visual system is built to do. Second, the experiments in this study do not measure the impact of environmental stability on the perception of environmental stability: Observers were not asked to explicitly rate shadow reliability, for example. The adopted protocols measure the impact of this manipulation on sensory discrimination of localized probes. The question of whether and how these two processes interact, and on what timescale they may do so, remains completely open.

To gain some insight into the above question, I repeated the sensitivity analysis separately for different epochs throughout each block: I applied a sliding window over the duration of each block and computed sensitivity values for each window separately (similar trends are visible for window sizes between 20 s and 2 min, except for inevitably noisier traces with shorter window durations). In this way, we can track how sensitivity changes throughout the 5-min duration of each block. The y-axis in Figure 3 plots the difference in sensitivity between image-based and object-based measurements: The upward shift between red and blue data clusters from Figure 2. As we have seen previously, this is the metric of interest for studying the impact of shadow manipulations on sensory measurements. We can therefore map Figure 2 directly onto Figure 3: Overlapping clusters (as in Figure 2A) correspond to near-zero values on the y-axis of Figure 3, while separated clusters (like those in Figures 2B–C) correspond to positive values in Figure 3. The important difference between Figure 2 and Figure 3 is that, with the latter, we can study how this effect evolves throughout the block.

Figure 3 shows that, at the beginning of the block (zero on the x-axis), the three shadow configurations produce similar results: The corresponding three traces show little difference (y values slightly above zero) between image-based and object-based insertions. As time progresses along the x-axis, the absent/unreliable-shadow configurations (orange/magenta) depart from the reliable-shadow configuration (black) to produce the data arrangement shown in Figure 2: separated red-blue clusters for absent/unreliable configurations (Figures 2B–C), corresponding to positive values for differential sensitivity on the y-axis in Figure 3 (trailing end of orange/magenta traces); overlapping clusters for the reliable-shadow configuration (Figure 2A), corresponding to near-zero values for differential sensitivity in Figure 3 (black trace). Notice that this transition occurs over a timescale of minutes: It takes approximately 2 min before the three traces separate to a substantial degree in this plot.

To assess the statistical reliability across observers of the effect demonstrated above, I split the data set into two halves: The early epoch, corresponding to the first half of the block (first 2.5 min), and the late epoch, corresponding to the second half of the block (remaining 2.5 min). Differential sensitivity values are not substantially different from zero during the early epoch (see confidence intervals around individual observer data for inset histograms on the left side of Figure 3, indicated by shaded regions), while they stay clear of the zero line for absent/unreliable-shadow configurations during the late epoch (orange/magenta insets on the right side of Figure 3). The results of this analysis support the observations made earlier in relation to the slow dynamics of the effects under scrutiny (notice that this analysis does not depend on the choice of sliding window adopted for the visualization in the main panel).

### Does image-based sensitivity increase, or object-based sensitivity decrease, or both?

A relevant question at this stage is whether the increase in differential sensitivity for the orange/magenta traces in Figure 3 reflects an increase in image-based sensitivity with no increase in object-based sensitivity, a decrease in object-based sensitivity with no decrease in image-based sensitivity, or a combination of these and/or similar effects. Answering this question is not straightforward, because absolute sensitivity varies greatly across observers and fluctuates substantially over the duration of a given block, most likely as a consequence of differences in task engagement, alertness, and attention. By computing differential sensitivity, these effects are largely factored out, making the differential measurement more robust/reliable. For example, different participants often produce very different patterns of sensitivity over the duration of the block: In one participant, sensitivity may decrease for both image-based and object-based cues over the duration of the block, while in another participant, both measurements may increase. However, the difference between image-based and object-based sensitivity may show similar patterns between the two participants.

For the above reason, when the data are analyzed at the level of individual observers (as in the insets to Figure 3), no clear trend emerges. If we ignore interindividual differences and only consider aggregate traces of sensitivity over time (such as those in the main panel of Figure 3), and if we measure time-dependence in the form of correlation between sensitivity and time, the following trends are suggested by the data: Image-driven sensitivity increases for the absent/unreliable-shadow configurations (correlation coefficients of 0.46/0.49) and decreases for the reliable-shadow configuration (correlation coefficient of −0.56); object-driven sensitivity decreases for the unreliable-shadow configuration (−0.91). The above trends return minuscule *p*-values (all <10^−6^) for a test of the null hypothesis of lack of correlation under normal distribution (see Kowalski [1972] for a discussion of various issues associated with this type of test), but we must be cautious in interpreting those values because they inevitably depend on the choice of sliding window adopted for computing the time traces, and they do not reflect significant structure at the level of individual observer analysis.

From the above, we must conclude that the data set associated with this study does not carry enough resolving power to conclusively answer the question of whether/how *absolute* sensitivity changes over time for image-based and object-based characteristics. Furthermore, even if one were to collect more data, it remains unlikely that this question may be answered decisively as a consequence of large fluctuations in engagement/attention across participants and over time for the same participant, if not by concluding that no consistent trend can be identified that adequately captures behavior at the population level.

### Qualitative model based on flexible cue combination

We currently lack sufficient knowledge to construct an explicit computational model of the observer performing sensory/memory tasks while freely navigating a complex environment. Even if we attempted to design such a model, it would require specification of so many parameters that the available data set would not be able to constrain it effectively. We can, however, restrict our efforts to a simplified model that may at least capture some important aspects of the experimental setting we are considering here. Furthermore, this model may support a type of data analysis that accounts for factors overlooked by the sensitivity/bias measurements. I illustrate one such factor below.

Consider the image in Figure 4A. This image shows a scene actually viewed by one of the observers during the experiments (as seen from inside the HMD). On this occasion, the probe was presented at the location indicated by the magenta circle. If one were to estimate the orientation defined by the environment at/near that location, the answer would be substantially different depending on the type of information that is used to carry out the estimate. If we construct an object-based map of the scene, the orientation defined by the environment around the location of probe insertion is near-vertical. This assessment is based on the presence of a clear object-based boundary defined by the box on the right (blue map/segments in Figure 4B) but runs in opposition to the indication provided by image-based information: On the basis of image information alone, and disregarding object structure, the orientation defined by the scene at/around probe location is predominantly horizontal (red map/segments in Figure 4B). On this trial, observers are therefore expected to produce substantially different responses depending on whether they rely on object-based or image-based information to make their judgment.

Consider now Figure 4I. This image shows a different scene. Here, the orientation defined by the environment at probe location is unmistakably horizontal, regardless of which information is used to assess it. When we generate object-based and image-based maps from the scene, they both assign horizontal orientation to that location (Figure 4J). On this trial, observers are therefore expected to produce the same response, regardless of which cue information (whether image-based or object-based) they use. As a consequence, trials of this kind are less informative about observer strategy than trials of the kind examined in the previous paragraph. When computing sensitivity values from the data, however, all trials are treated in the same way, without differentiating between those that are more similar to Figure 4A and those that are more similar to Figure 4I. Furthermore, the sensitivity calculations do not take into account trial-by-trial fluctuations associated with orientation noise within the probe (see Methods): On different trials, the physical orientation energy carried by the probe may differ substantially from its target orientation (aligned with, or orthogonal to, the congruent orientation). This trial-by-trial variation must impact the sensory task to varying extents, but it is entirely overlooked by the sensitivity calculation.

To address the above issues, and possibly use them as starting point for gaining further insight into the strategy adopted by observers, I implemented a simple model that essentially retraces the logic laid out in previous paragraphs. The model operates in three steps: First, it estimates local orientation from the noisy probe presented on a given trial; second, it estimates the congruent orientation defined by the scene on the same trial; and third, it compares the two estimates to make a determination as to whether the probe is congruent or incongruent on that specific trial. These steps rely on three actual snapshots of the scene, taken on every trial during the experiments. One snapshot (Figure 4F) is used to estimate the absolute orientation of the local region occupied by the probe (Step 1 above), without reference to whether it may be congruent or incongruent. The remaining two snapshots (one is shown in Figure 4A) are used to construct image-based and object-based maps for the scene, as it appeared to observers immediately preceding probe presentation (see Methods for details). The model must then produce an estimate of “congruency” (Step 2 above) to serve as reference orientation against which to evaluate the orientation of the probe (Step 3). This estimate is constructed by combining separate estimates from image-based and object-based maps (Figure 4C). The weighted combination is controlled by factor *w*: When *w* = 0, only object-based information is used to define “congruent” (vertical blue segment on the left of Figure 4D); when *w* = 1, only image-based information is used (horizontal red segment on the right of Figure 4D). For a given value of *w*, the model proceeds to define an estimate for the congruent orientation and compares it against the estimate obtained from the probe. Based on this comparison, it produces a binary congruent/incongruent response (Figure 4D) that is directly comparable with those produced by human observers (Figure 4G).

We can then fix all model parameters except *w* (see Methods) and identify *w* values that maximize the match between human and model responses on a trial-by-trial basis (agreement). Figure 4H shows that, as *w* varies along the x-axis, the corresponding human–model agreement (y-axis) for the reliable-shadow configuration (black trace) reaches a peak for *w* < 0.5 (to the left of vertical green line), indicating that slightly more weight is allocated to object-based cues compared with image-based cues. In the absent/unreliable-shadow configurations, the optimal *w* values are >0.5 (orange/magenta traces peak to the right of vertical green line), indicating that more weight is allocated to image-based cues. These results are broadly consistent with those obtained from the sensitivity/bias measurements (Figure 2; see next paragraph for further consideration of specific features that do not conform to this statement). At the same time, they offer a more tangible interpretation for those effects in terms of weight allocation to different sources of visual information. If we subscribe to the model depicted in Figure 4, we may interpret shadow availability/reliability as a higher-level factor controlling *w* and therefore adjusting perceptual reliance on image-based versus object-based cues in the scene.

The location of the peak in human–model agreement for the reliable-shadow configuration (black trace in Figure 4H), which is shifted toward greater weight on object-based cues (*w* < 0.5), may lead to the reasonable expectation that the model should display greater object-based sensitivity than image-based sensitivity for this configuration. This pattern would be inconsistent with the human measurements, which do not show significant differences between image-based and object-based sensitivity (Figure 2A). The connection between *w* and model sensitivity is, however, not straightforward, in the same way that the connection between *w* and human–model agreement is not obvious: Many factors contribute to this connection, such as trial-to-trial variations in the reliability of image-based versus object-based cues, response bias, and differences in the efficacy of the selected model parameters for extracting the two cues. For example, even though equal weight may be allocated to the two cues (*w* = 0.5), object-based cues may be noisier than image-based cues, so that object-based sensitivity may be lower than image-based sensitivity. Under this scenario, *w* = 0.5 would not transparently translate to equal sensitivity for the two cues. In the simulations, the model sensitivity values associated with peak human–model agreement were virtually identical for the reliable-shadow configuration, differing by less than 1% between cues. For the other two shadow configurations, image-based sensitivity was 40%/58% (absent/unreliable) higher than object-based sensitivity. These results mirror the human measurements (Figure 2) and provide a useful illustration of the difficulties involved in establishing a transparent connection between specific cognitive constructs, such as cue weight allocation, and final behavioral metrics, such as sensitivity. In a sense, those difficulties justify modeling exercises such as the one presented here, because they equip the formulation of cognitive theories with more tangible tools that can be tested and evaluated in software.

Notwithstanding the utility of implementing simulated observers of the kind exemplified by the tools in Figure 4, I emphasize that the model proposed above is not really a model, for several reasons. First and foremost, it does not provide a mechanistic account of how shadow information is incorporated into scene reconstruction or, for that matter, of scene reconstruction itself. It merely produces *w* values that project to trial-by-trial responses in close vicinity of the human data. In this sense, it represents little more than a fitting exercise, rather than a computational model with genuine explanatory power. An additional limitation comes from the many arbitrary choices that went into designing specific steps, such as the spatial extent over which map information is pooled to generate an orientation estimate for the congruent reference. For these reasons, the proposed model should not be regarded as a critical component of the conclusions we draw from the data or as an instrument for adding genuine depth of understanding to this study. It should be intended in the spirit of an interpretational framework for illustrative purposes. In essence, it amounts to rephrasing the intuitive terminology adopted earlier (flatness/depthness, object-based→image-based shift) using more tangible concepts.

### Memory task and sensory task interact in complex ways

The focus of this study is centered primarily on the sensory task: Our goal is to understand whether and how sensory processing may be impacted by complex behavioral phenomena that are not normally understood as relevant to the acquisition of proximal sensory information. In this context, the memory task does not play a critical conceptual role, but it does fulfill an important practical role: It prompts observers to engage in active reconstruction of scene layout. For this reason, not only observers were asked to perform two tasks at the same time, but additional task rules were specifically designed to ensure that ignoring one task would impact performance in the other task (see Methods).

Notwithstanding our focus on the sensory task, the memory task equally supports an explicit measure of performance (percentage of correct responses in a two-alternative forced choice). At the same time, it comes with a much smaller data mass: For every block, I recorded 100 measurements from the sensory task and only 1 measurement from the memory task. Because of this sizable reduction in data mass, we cannot analyze results from the memory task with the same level of detail afforded by the sensory task. More specifically, we can only examine values aggregated across observers, with no insight into interobserver variability/consistency. Notwithstanding this limitation, we observe internally consistent patterns that also present interesting peculiarities.

Figure 5A plots the percentage of correct box choices for the three different shadow configurations. We first notice that performance never reaches ceiling. This result provides direct evidence that the memory recall task was sufficiently difficult to require active engagement on the part of observers, as it was specifically designed to do. Perhaps surprisingly, performance was at chance (50%) for this task in the reliable-shadow configuration, while it reached threshold values around 75% for the absent/unreliable-shadow configurations. In the Discussion, I propose an interpretation of this finding based on prior results from the VR literature.

A relevant question at this stage is whether the above-documented interaction between shadow configuration and task performance is determined by these two factors alone, or whether there may be more complex effects at play. For example, we may query the role of the sensory task: What happens when observers are not engaged in this task, and therefore able to devote their entire efforts to the memory task? To answer this question directly, I repeated the experiments in the absence of the sensory task: Probes were removed altogether, and observers were only asked to identify which box moved at the end of each block. Because the memory task can be performed more easily under these conditions, it was necessary to reduce block duration substantially (below 1 min) to achieve threshold performance (see Methods). The resulting measurements show no difference across shadow configurations (open symbols in Figure 5A): Performance in the reliable-shadow configuration recovers to the level measured for the other two configurations. It therefore appears that the sensory task can impact the memory task in complex ways, by only affecting configurations with reliable shadow information.

The above results indicate a measurable impact of the sensory task on the memory task. We may wonder about the opposite direction: Does performance in the memory task impact the sensory task? We can attempt to answer this question by splitting blocks into those for which observers produced a correct recall response and those for which the recall response was incorrect. When comparing different shadow configurations following this split, however, we find mismatched data mass for the different configurations: Because the reliable-shadow configuration was associated with chance performance, the split is roughly even, while the other two shadow configurations are associated with uneven splits of 3/4 to 1/4 (correct to incorrect). To ensure that data mass is more appropriately matched when comparing shadow configurations, I combined absent-shadow and unreliable-shadow configurations into one configuration (absent/unreliable).

Figures 5B–C plots differential sensitivity for both absent/unreliable- and reliable-shadow configurations. As expected, this metric is near zero for the reliable-shadow configuration (black elements in Figures 5B–C), and positive for the absent/unreliable configuration (cyan elements). More importantly with regard to the specific question we are presently asking, this difference persists regardless of performance in the memory task: Estimates in Figure 5B, obtained from blocks on which observers responded correctly to the memory task, are virtually identical to those in Figure 5C, obtained from blocks on which observers responded incorrectly. We therefore find no connection between success/failure in the memory task and shadow effects in the sensory task. A more direct approach to exploring the potential impact of the memory task on the sensory task would involve complete removal of the memory task. Unfortunately, this manipulation would severely alter the general exploratory behavior engaged by observers in the arena (see Methods for a detailed explanation), which would in turn make it impossible to obtain interpretable measurements from the sensory task.

## Discussion

The most significant result of this study is the demonstration that complex, long-term environmental statistics is implicitly incorporated into early sensory processing by freely navigating human agents. In this context, early sensory processing refers to visual extraction of elementary features, such as edges and bars in the scene (Marr, 1982; Morgan, 2011). As a consequence, the results presented here pertain to the point of contact between two extremes of sensory behavior: on the one hand, immersive exploration of 3D environments for spatial retrieval of scene layout; on the other hand, local analysis of the retinal input for proximal image reconstruction. Below I elaborate further on this result, its connections with existing literature, and its implications for understanding sensory processes during natural behavior.

Before embarking on a detailed discussion of specific issues and relevant studies, I address the theoretical motivation behind my choice of experimental design. Perhaps the most obvious question in relation to the adopted paradigm is: Why shadows? There are many other environmental characteristics that one may choose to manipulate, such as spatial consistency (Warren, 2019; Muryy & Glennerster, 2021) or texture composition (Isoyama, Sakuragi, Terada, & Tsukamoto, 2021). My motivation for manipulating shadows stems from the pivotal role played by this scene component in formulating early theories of biological image reconstruction (Gibson, 1979; Marr, 1982). Shadows perfectly exemplify the difficulties involved in successfully segmenting natural images (Neri, 2017): On the one hand, they cast sharp edges across the scene, thus generating salient features that vigorously drive local edge detectors (Morgan, 2011); on the other hand, they do not directly inform the visual system about fundamental properties of the environment, such as object boundaries (Bansal, Kowdle, Parikh, Gallagher, & Zitnick, 2013).

In a sense, the most burdensome job for the early visual system is to sort out edges into whether they are structural (associated with genuine stable properties of the environment) or incidental (generated by transitory factors such as shadow casting). This is not to say that shadows are useless for inferring environmental structure — to the contrary, they can provide important information about 3D shape and other properties of the scene (Kersten, Mamassian, & Knill, 1997; Mamassian et al., 1998; Dee & Santos, 2011). The process by which this information is inferred from shadows, however, cannot be merely characterized as detection and involves more complex knowledge about the environment (Gibson, 1979; Cavanagh, 1991). From the viewpoint of early extraction of elementary features from the proximal image (Morgan, 2011), shadows (and more generally image rendering under different lighting conditions) pose serious challenges for recovering scene structure (Rensink & Cavanagh, 2004). When it comes to shadows, these challenges are encountered all the time during natural vision. For the above reasons, shadows have played a dominant role in early thinking about biological vision from a computational standpoint (Marr, 1982), and they represent a natural tool for interrogating the higher-level properties of scene reconstruction. These considerations have shaped the design of the experiments.

In approaching existing literature of potential relevance to this study, I notice that (to my knowledge) no prior study has attempted to characterize early vision via low-level psychophysical measurements while observers actively engage in virtual scene exploration. However, several existing studies have examined either one of those two aspects of sensory processing. Below I select and discuss studies that are most directly relevant to the present one.

Previous work has provided useful knowledge about detection/discrimination of localized wavelets in the presence of complex contexts, such as those experienced during natural vision. If we adopt the terminology introduced earlier, existing evidence may be characterized in the following terms: detection is primarily determined by image-based cues (Sebastian, Abrams, & Geisler, 2017), while discrimination is primarily determined by object-based cues (Neri, 2017) (with some exceptions; Teufel, Dakin, & Fletcher, 2018). For example, efficiency for detecting targets/distortions within local regions of natural scenes can be satisfactorily accounted for on the basis of low-level image properties (Sebastian et al., 2017) such as edge contrast (Bex, Mareschal, & Dakin, 2007), density (Bex et al., 2009), and phase (Rideaux, West, Wallis, Bex, Mattingley, & Harrison, 2022). When observers are required to engage in orientation discrimination for determining congruency, however, other factors come into play, many of which are not directly connected with low-level image-based content (Neri, 2017). These factors range from those associated with object segmentation (Neri, 2017) to those impacted by semantic interpretation (Neri, 2011; Neri, 2014). At the same time, not all results fall into the dichotomy above, which inevitably provides an oversimplified picture of current literature. To provide one example, detection of oriented wavelets inserted into degraded scenes may improve following acquisition of higher-level knowledge about the scene, despite no change in physical content of the image (Teufel et al., 2018).

At least superficially, the adopted sensory task is comparable with previous orientation discrimination tasks involving a congruent/incongruent determination (Neri, 2014; Neri, 2017). In prior experiments with static natural scenes, performance was superior for object-based probe insertions compared with image-based insertions (Neri, 2017). I do not measure this effect here: Performance for object-based insertions is at best equivalent to that for image-based insertions (in the reliable-shadow configuration, Figure 2A). Although I do not have a ready explanation for this apparent discrepancy, I must emphasize the innumerable differences between the conditions of prior experiments and those used here. In addition to differences relating to scene appearance, spatial uncertainty, timing, and other stimulus-specific parameters, the major difference clearly relates to the VR medium. Previous studies involved flat static natural scenes flashed in front of fixating observers (Neri, 2017) and are therefore not comparable with the experiments described here. Even if one were to hypothesize that the underlying perceptual processes should behave similarly, the VR implementation involves technical difficulties that make it impossible to compare measurements across studies. More specifically, probe alignment can be substantially inaccurate on some trials, and the minimal requirements for satisfactory insertion (see Methods) are sometimes met only partially in real time. These technical glitches do not apply to experiments with preprocessed images outside VR (Neri, 2014; Neri, 2017), and they do not necessarily impact image-based insertions to the same extent that they impact object-based insertions. As a consequence, it is impossible to make confident predictions about the relationship between performance within VR and performance outside VR (Draschkow, Nobre, & van Ede, 2022).

An iconic result in the VR literature with potential connections to the paradigm adopted here comes from early studies in which agents were asked to complete specific tasks involving simple objects, such as blocks of different shape and color (Ballard, Hayhoe, & Pelz, 1995). Overall, this line of research has established that perception happens “online,” in the sense that observers do not constantly engage in detailed reconstruction of the scene, but only do so when they are asked to take specific action in relation to a given aspect/portion/element of a scene (Hayhoe, Bensinger, & Ballard, 1998). As a consequence, they may overlook substantial changes in the physical structure of the scene, when those changes do not fall within their goal-oriented focus (Triesch, Ballard, Hayhoe, & Sullivan, 2003). In other VR experiments, observers may fail to notice large changes in the surrounding environment, such as rooms undergoing implausible transformations (Glennerster, Tcheang, Gilson, Fitzgibbon, & Parker, 2006), or they may disregard gross inconsistencies in the spatial layout of a virtual maze (Muryy & Glennerster, 2021; Warren et al., 2017). Results of this kind appear to share some broad characteristics with the results presented here, but it is not easy to establish a clear connection between the two sets of studies (see below).

On the one hand, the VR findings reviewed above remind us of the extent to which scene perception is about gist rather than detail (as also demonstrated by the phenomenon of change blindness; Simons & Levin, 1997; Triesch et al., 2003). In this sense, they emphasize the persistent connection between low-level and higher-level processes in perception, a result that aligns well with those presented here. In particular, they may provide a tentative explanation for the decrease in recall performance associated with the sensory task in the reliable-shadow configuration (solid vs. open black symbols in Figure 5A): When observers are required to take action with relation to specific regions of the scene to determine probe congruency, their strategy for memorizing room layout may shift from a detail-oriented representation toward a gist-oriented representation of the scene, thus freeing up resources for detail-oriented processing of the local region occupied by the probe. This shift in strategy would lead to reduced performance in the memory task (under the assumption that a gist-based map of the room would not support accurate identification of box displacements) and would be broadly consistent with the perceptual theory that has emerged from previous work in VR (Ballard et al., 1995; Triesch et al., 2003). However, this account does not explain the different results obtained for different shadow configurations, unless we introduce further interpretational elements. For example, we may hypothesize that gist-oriented encoding of the scene is not engaged when environmental statistics is violated by implausible manipulations, thus preventing the shift from detail-oriented to gist-oriented representation when shadow statistics is absent/unreliable. This interpretation, however, remains highly speculative, with little direct evidence in the data to support or exclude it.

On the other hand, those same VR findings indicate that the long-term statistical consistency of the environment is largely ignored by observers (Ballard et al., 1995), which appears at odds with the present finding of measurable differences between reliable- and unreliable-shadow configurations (although see Hayhoe et al. [1998] for evidence of overlapping processes operating at different timescales). Those differences must reflect the long-term statistical degree of reliability associated with shadow information, because the two shadow configurations did not differ in any other respect. More specifically, on the short timescale (∼200 ms) of the sensory task from which the sensitivity measurements are derived, there is no difference in environmental statistics between these two configurations. It is important to recognize, however, that observers were never asked to make explicit judgments about changes in the environment: The sensitivity measurements reflect long-term statistics implicitly, via their effect on sensory performance in the probe discrimination task. In other words, they reflect a form of implicit statistical leaning (Perruchet & Pacton, 2006). This is an important difference with respect to classic VR studies in which observers were explicitly asked to report on potential changes in the appearance of objects within the environment (Ballard et al., 1995; Triesch et al., 2003), and may explain the apparent inconsistency between their results and those reported here. For example, it is conceivable that in the present experiments, observers may not register environmental changes to the extent of being able to describe them in explicit detail (Nightingale, Wade, Farid, & Watson, 2019), and that asking them to do so may reset their implicit adaptation to said changes.

The timescale associated with the perceptual accrual of shadow information and its impact on sensory processing, which extends over minutes (Figure 3), may seem implausibly long. The nature of this finding is particularly surprising in consideration of the fact that the primary metric of differential sensitivity relates to the sensory task, which completes on the subsecond timescale. This result, however, is reminiscent of early findings on storage of aftereffects: Under some conditions, a few minutes of exposure to the adapting stimulus may produce measurable aftereffects 3 months later (Jones & Holding, 1975; Favreau, 1979). These findings are often overlooked by contemporary literature, because they are difficult to reconcile with modern notions of sensory adaptation. The present experiments differ in many respects from those carried out to test aftereffects and related phenomena, but they share the same surprising characteristic of demonstrating interactions across perceptual processes that may seem incommensurable according to mainstream views of perception.

I propose a novel conceptual framework for understanding sensory adaptation. In this framework, which shares numerous features with Gibsonian notions of ecological perception (Gibson, 1979; Warren, 2021), sensory recalibration is driven by the ecological structure of environmental statistics. This structure must be understood in the broadest sense to incorporate various aspects of how the physical environment behaves and how it interfaces with the perceptual systems of the observer (Gibson, 1979). Furthermore, it is not just structure that matters, but also the statistical reliability associated with subelements of this structure. In the present experiments, for example, it is not only relevant whether shadows are consistent or inconsistent with natural everyday experience: Their reliability (or lack thereof) also plays a role in structuring their impact on perception. At this stage, we do not hold a complete account of how the representation of this environmental “Umwelt” affects perception. The experiments in this study provide quantitative evidence that it exists and offer specifics on how it operates with regard to shadow statistics. More importantly, these results emphasize the highly sophisticated nature of the underlying processes and their pervasive reach into perception at all levels (whether early or late) and scales (whether local or global, whether fast or slow). Future experiments will be necessary to explore and characterize the full extent of this complex phenomenon.

## Supplementary Material

Supplement 1

Supplement 2
