# Supplementary material for: Human sensory adaptation to the ecological structure of environmental statistics
Source: J Vis. 2024 Mar 5;24(3):3. doi: 10.1167/jov.24.3.3 (PMC10916885; doi:10.1167/jov.24.3.3)
Supplement: Supplement 1 [file jovi-24-3-3_s001.pdf]

Supplementary Material for: Human sensory adaptation to the ecological structure of environmental statistics

Peter Neri
Laboratoire des Systèmes Perceptifs (UMR8248), École normale supérieure, PSL Research University
29 rue d'Ulm, Paris 75005, France

Keywords: virtual reality, object segmentation, statistical learning

Differential role of sensitivity and response bias

Supplementary Figure 1A plots differences in sensitivity (black symbols) and criterion (green symbols) between image-driven and object-driven conditions, for the absent shadow configuration (x axis) versus the unreliable shadow configuration (y axis). The coordinates of these data points are directly connected with those plotted in **Figure 2**: black symbols in **Supplementary Figure 1A** plot differences in the y coordinate between red and blue data points in **Figure 2**, while green symbols plot differences in the x coordinate. In line with **Figure 2**, black symbols in **Supplementary Figure 1A** are associated with larger absolute values than green symbols: the effect size associated with d′ estimates is substantially larger than the effect size associated with c estimates, indicating that the object-driven→image-driven shift is attributable to sensitivity changes, not changes in response bias. The small effect observed for c estimates (green symbols tend to fall within the lower left quadrant) is most likely caused by a sensitivity change that spills over onto the bias estimate, because of imperfect separation of the underlying phenomena (see Methods).

The above conclusion is corroborated by the comparison between estimates from different shadow configurations. If a given metric reflects genuine cognitive effects, we expect that this metric should show consistent inter-individual differences: if participant X produces smaller changes than participant Y when they are both tested in the absent shadow configuration, we expect that the same relationship (smaller changes for X compared with Y) should be measured when they are tested in the unreliable shadow configuration. **Supplementary Figure 1A** demonstrates that this relationship only applies to d′ estimates (black symbols are significantly correlated with Pearson coefficient r=0.72 and p=0.01), not to c estimates (green symbols are not significantly correlated with p=0.79). **Supplementary Figure 1B** plots correlation coefficients for all three pairwise comparisons between shadow configurations (leftmost values refer to plot in panel **A**). It is clear that sensitivity estimates present consistent structure (black symbols are greater than 0, indicated by horizontal dashed line), while criterion estimates lack such structure (green symbols fall around 0).

From the above, we cannot exclude that criterion estimates carry information about the object-driven→image-driven shift: the correlation may be present, but too small/noisy to measure. Indeed, these estimates most likely do carry relevant information as a consequence of the spill-over from sensitivity discussed above. However, we can certainly conclude that the bulk of relevant information about the object-driven→image-driven shift is carried by d′ estimates, and most importantly that this shift must be attributed to changes in perceptual sensitivity, not response bias.

doi: Received: December 22, 2023 ISSN 1534–7362 © 20?? ARVO

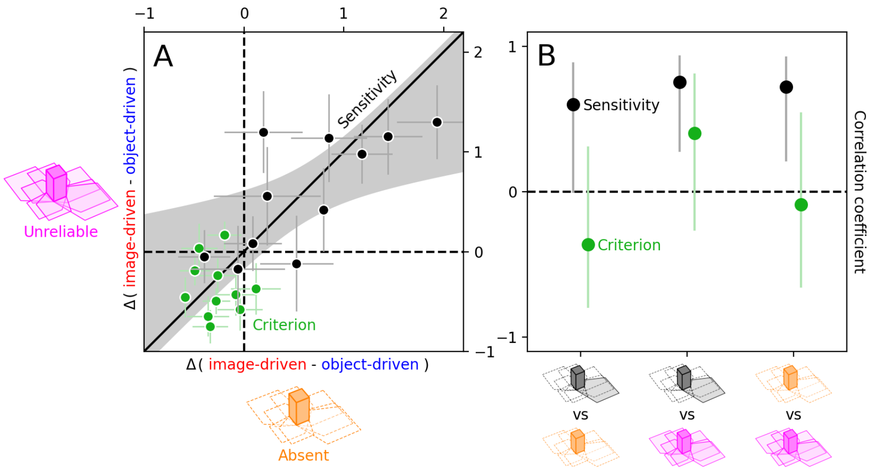

Figure 1: *

Supplementary Figure 1: Object-driven→image-driven effects reflect sensitivity shifts, not criterion shifts. A plots differences (Δ) in sensitivity/criterion (black/green symbols) between image-driven and object-driven insertions for the unreliable shadow configuration (y axis) versus the absent shadow configuration (x axis) across observers (one data point per observer). ΔSensitivity values are significantly correlated between shadow configurations (shaded region shows 95% confidence intervals around linear fit), while Δcriterion values are not (see main text for statistics). This result applies to correlation coefficients from all three pairwise comparisons between shadow configurations in **B**: reliable versus absent (left), reliable versus unreliable (middle), absent versus unreliable (right). Error bars indicate ±1 SEM in **A**, and 95% confidence intervals in **B**.

Distribution of probe/eye/head/body across display and room spaces

Probe sampling

The probe insertion algorithm (detailed above) did not enforce uniform sampling of the visual field, because this constraint made it impossible to identify viable insertion points on the fly. As a consequence, probe sampling differed substantially between object-driven and image-driven insertions. More specifically, image-driven insertions were biased towards the upper visual field (red pixels/histograms in **Supplementary Figure 2A–C**), particularly in the absent shadow configuration (**Supplementary Figure 2B**). The implications of this bias for sensitivity estimation are not straightforward, which complicates a direct comparison of this metric between image-driven and object-driven insertions. However, the results show virtually identical degrees of bias across shadow configurations. It is therefore expected that, whatever the impact of this upward bias may be, it does not invalidate comparisons across shadow configurations, which represent the focus of interest for the present study.

Head location/direction and eye position

I recorded the 3D position and direction of the HMD from all experimental sessions (indicated by green arrows in **Supplementary Figure 2E–G**), and performed several comparisons across shadow configurations to identify potential differences in head movement statistics. I was not able to identify any such difference based on the distribution of various parameters such as direction, velocity, frequency of head turns, range of room exploration, and several other related characteristics. For a subset of the sessions, I was also able to acquire eye position information. I assessed the reliability of the integrated eye-tracker provided by the HTC Vive Pro Eye during independent tests in which I placed small objects at the tracked position, and compared their placement with eye movements to different objects within a virtual room. These tests indicated that, although eye-tracking was acceptable with respect to direction of gaze, vergence measurements were unsatisfactory for the purposes of this investigation. I therefore report only direction of gaze in **Supplementary Figure 2E–G**. In general, I found that observers maintained their fixation near the center of the visible display (black dots/histograms

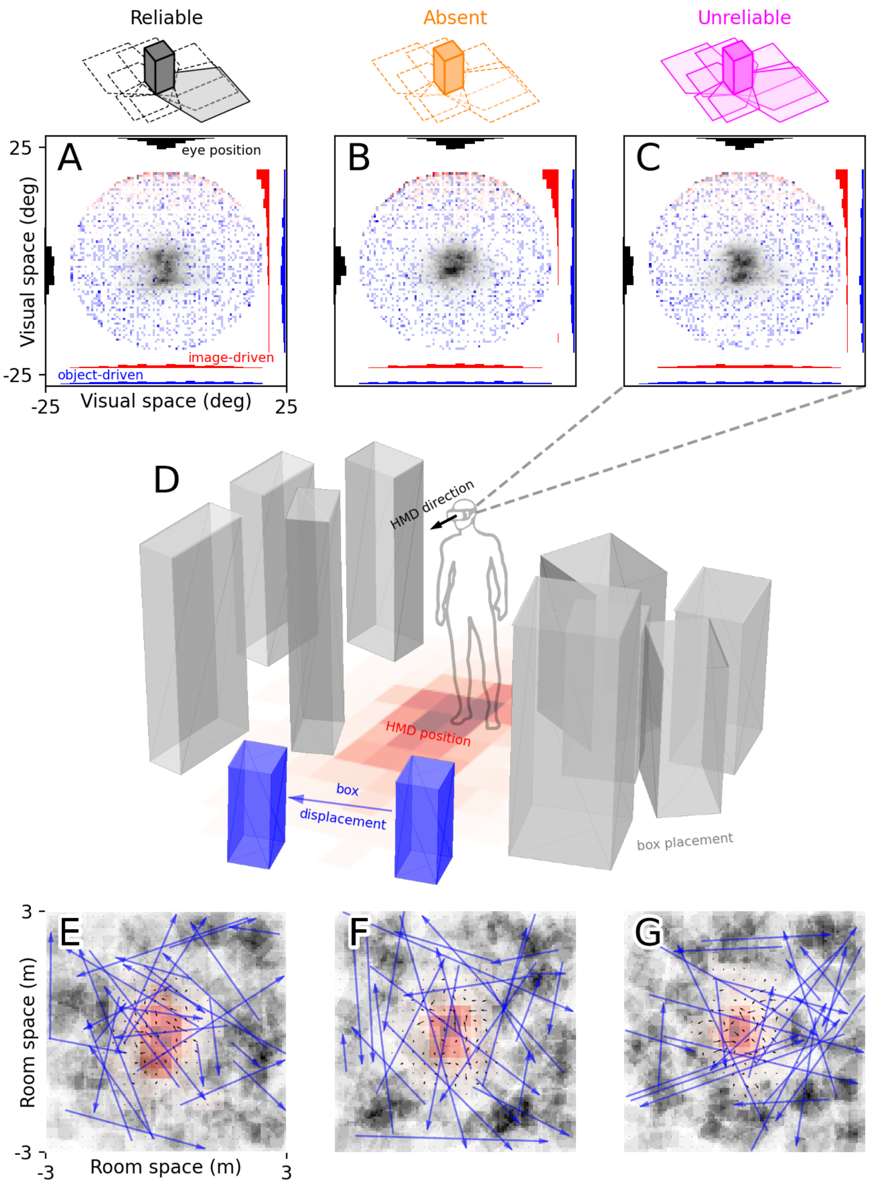

Figure 2: *

Supplementary Figure 2: No measurable differences across shadow configurations with respect to probe distribution, eye movements, head movements, or box placement/displacement. Red/blue dots in **A** show probe locations for image-driven (red) and object-driven (blue) insertions across the HMD display in the reliable shadow configuration. Black dots show points of fixation from eye-tracking. Red/blue/black histograms show corresponding distributions across both axes. **B**–**C** plot equivalent information for absent and unreliable shadow configurations. **E** plots 2D distribution of HMD locations across the arena (red tint with greater saturation indicating higher frequency), average HMD pointing direction for different positions within the arena (green arrows with arrow length reflecting relative frequency), box distribution across the room (gray tint with saturation reflecting greater frequency of box placement), and box displacements during teleportation for memory task (blue arrows indicating individual box displacements, one for each block of data collection). **D** provides a pictorial legend for interpreting **E**. **F**–**G** plot equivalent information for absent and unreliable shadow configurations.

in **Supplementary Figure 2A–C**): room exploration was almost exclusively achieved via head movements, not eye movements. As for head movements, I was not able to identify any substantial difference across shadow configurations.

Supplementary Video: Example experiment as seen from inside the HMD

Observers were initially placed inside a white room with logos of the game they were asked to play, which I named "Room Recall." After pressing a button to initiate the experimental block, the room was populated with boxes and lined with zigzag patterns. This video shows a few examples of probe insertions at various locations, followed by feedback (green/red disc in the centre of the display). The actual experience in virtual reality is substantially different from viewing this video, so the video is only intended for illustrative purposes to provide a general impression of the room as seen by participants.
